# Supplementary material for: Systematic assessment of microenvironment-dependent transcriptional patterns and intercellular communication
Source: Genome Biol. 2025 Jul 6;26:193. doi: 10.1186/s13059-025-03677-5 (PMC12232842; doi:10.1186/s13059-025-03677-5)
Supplement: Supplementary file 1 — Additional file 1: Supplementary Figures. Figures S1 to S34. [file 13059_2025_3677_MOESM1_ESM.pdf]

Supplementary figures

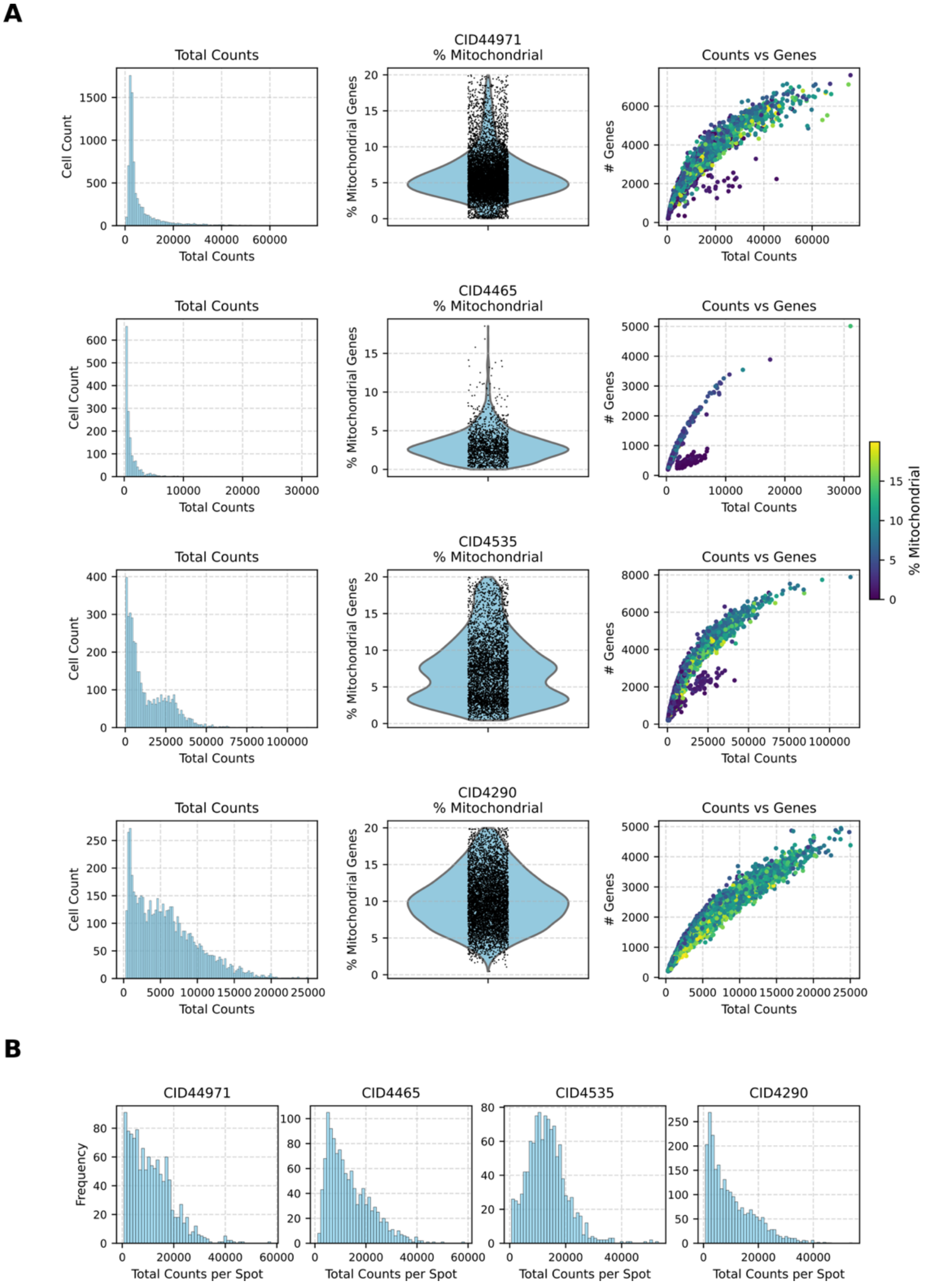

Fig S1. Quality control (QC) metrics for scRNA-seq and spatial transcriptomics data from the Breast Cancer dataset by patient. **A**. QC results for scRNA-seq data for patients CID44971, CID4465, CID4535, and CID4290. For each patient we represent the distribution of total UMIs (counts per cell), a violin plot showing the percentage of mitochondrial gene content and a scatterplot to illustrate the relationship between total UMIs (x-axis) and detected genes per cell (y-axis), colored by mitochondrial gene percentage. **B**. Distribution of total UMIs per spatial spot for the same patients.

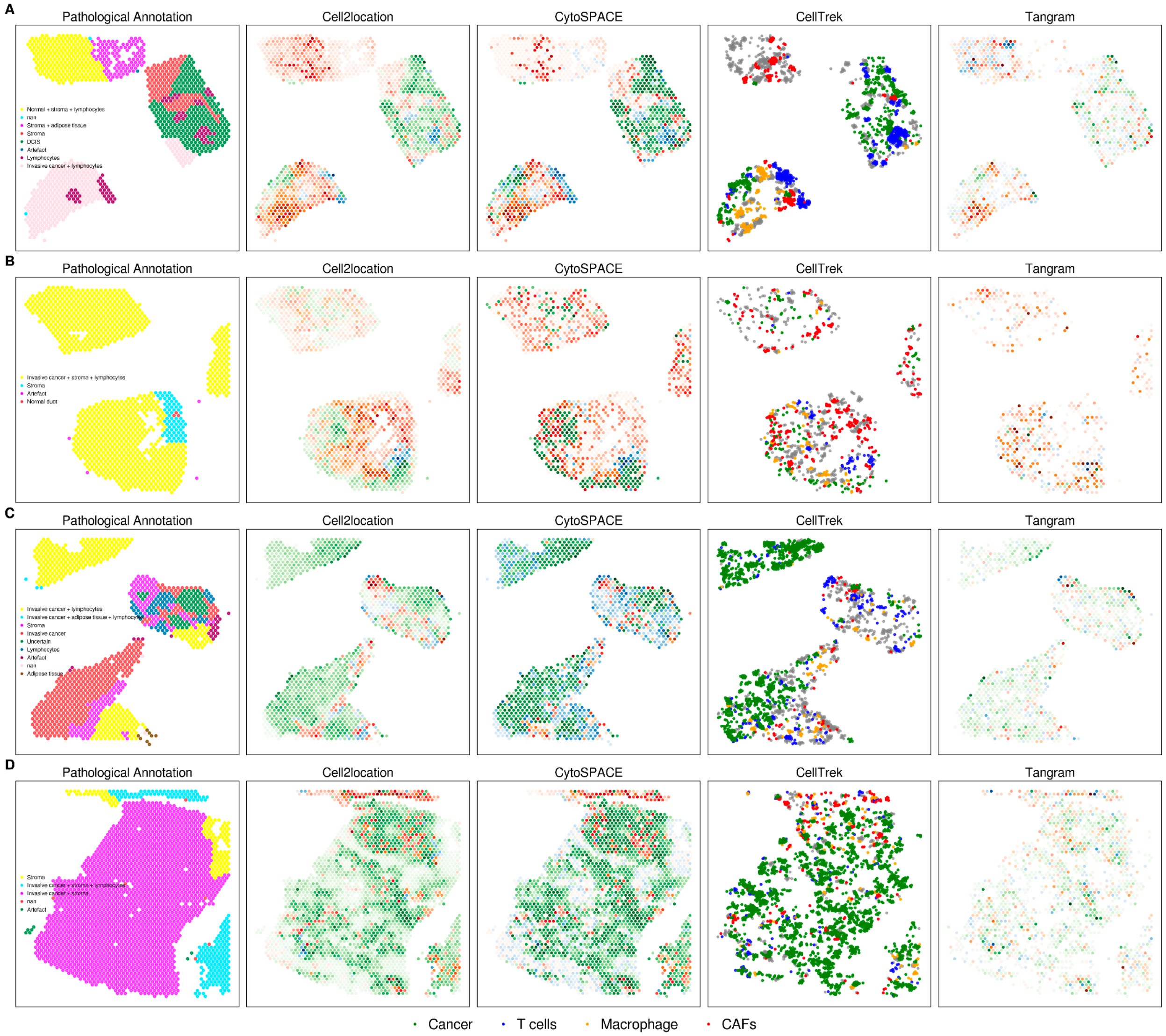

Fig S2. Methodological comparison for disentangling single-cell spatial resolution for breast cancer dataset. We applied four methodologies - Cell2location, CytoSPACE, CellTrek and Tangram - to determine cell positioning across different patients (identified as CID44971, CID4465, CID4535, CID4290, and labeled A, B, C, D respectively). Pathological annotations from the original publication are included for comparative analysis (Wu et al., 2021), showing notable concordance between the methodologies and the annotations. The figure illustrates the normalized abundance of predominant cell types, with each spatial spot representing the cell type of highest abundance for clear visualization. Color coding: green cancer cells, blue T cells, red macrophages, orange Cancer Associated Fibroblasts (CAFs) and grey the rest of the cell types.

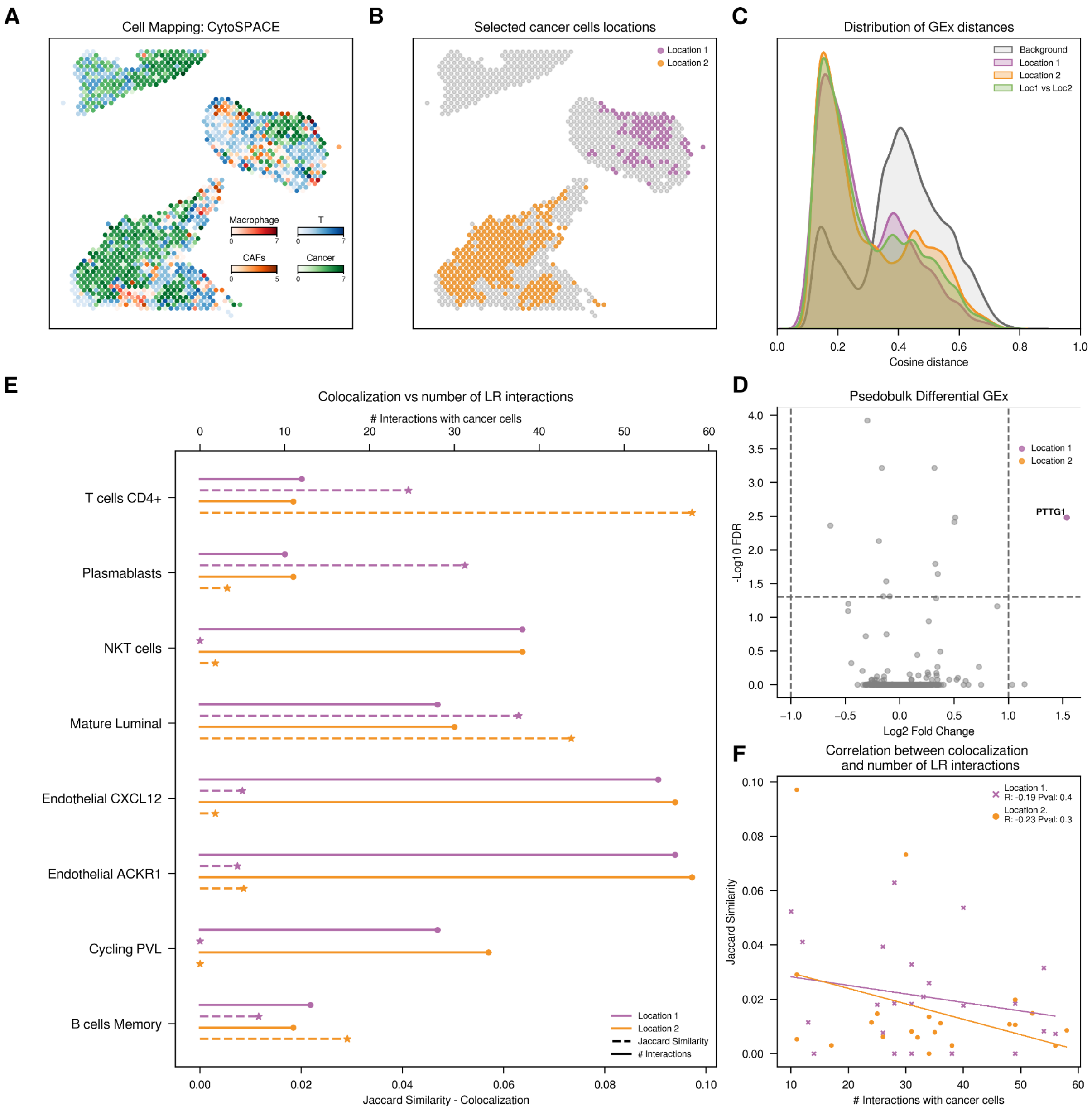

Fig S3. Exploration of cancer cells from CID4535 patient (bottom-middle). **A**. Distribution of main cell types across the tissue slide. For visualization purposes, we normalized the CytoSPACE inferred abundances of predominant cell types, with each spatial sport representing the cell type with highest abundance. **B**. Spatial representation of cancer cells according to their assigned location. **C**. Distribution of cosine distances between single-cell gene expression (GEx) profiles. We analyze the cosine distances between gene expression patterns of cancer cells located within the same or different tissue regions. **D**. Volcano plot with Differential Gene Expression results, highlighting significant upregulated genes in cancer cells of each region. **E**. Comparison of colocalization and cell-cell communication results. Plain line (top axis) represents the count of significant ligand-receptor interactions between each of the defined cancer cells and the y-axis cells. Dotted line (bottom axis) indicates colocalization, measured by the Jaccard similarity index based on the presence or absence of cells within each spot. **F**. Spearman correlation between colocalization and ligand-receptor counts. Color code: violet cancer cells in location 1, orange cancer cells in location 2.

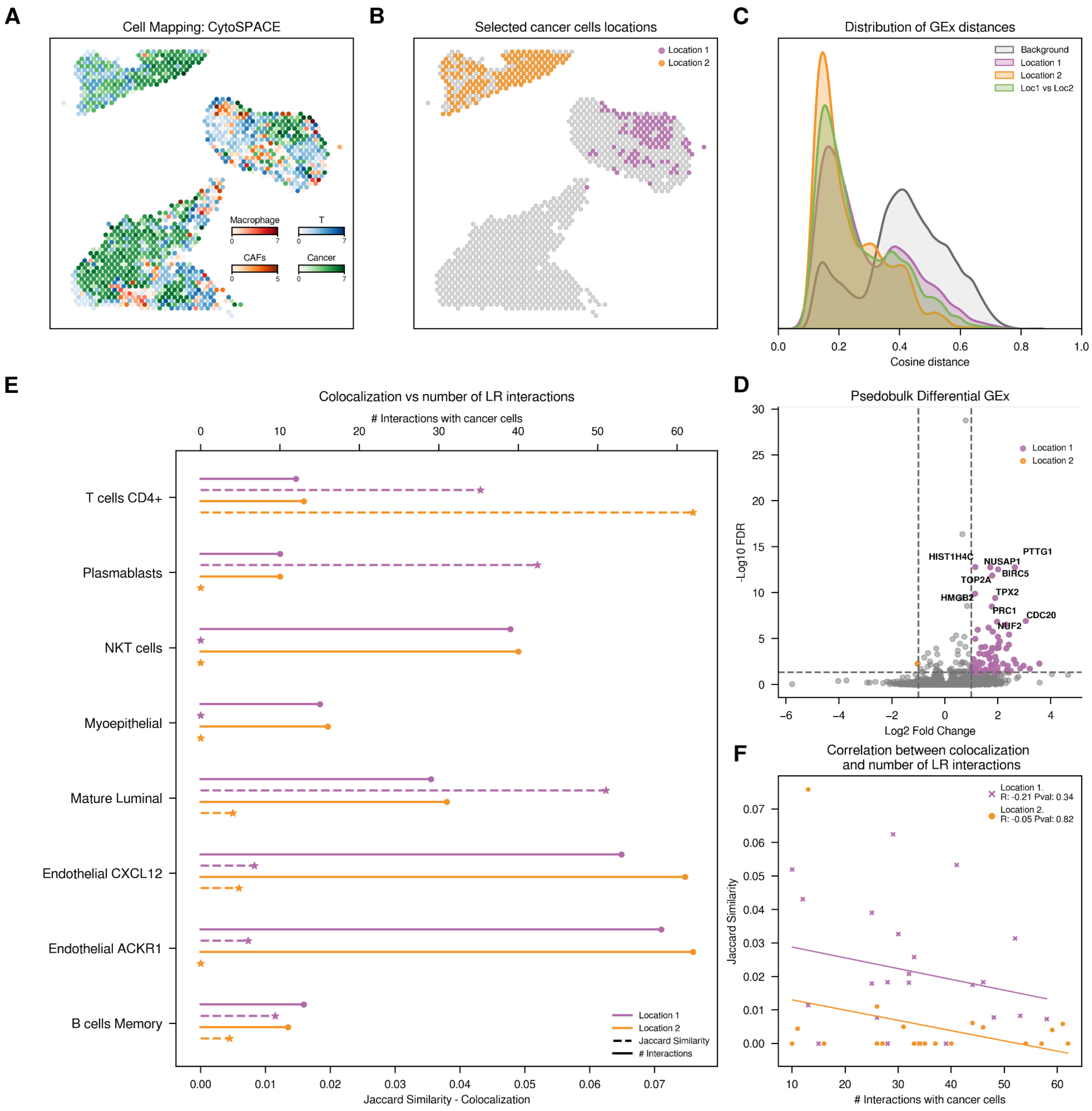

Fig S4. Exploration of cancer cells from CID4535 patient (top-middle). **A**. Distribution of main cell types across the tissue slide. For visualization purposes, we normalized the CytoSPACE inferred abundances of predominant cell types, with each spatial sport representing the cell type with highest abundance. **B**. Spatial representation of cancer cells according to their assigned location. **C**. Distribution of cosine distances between single-cell gene expression (GEx) profiles. We analyze the cosine distances between gene expression patterns of cancer cells located within the same or different tissue regions. **D**. Volcano plot with Differential Gene Expression results, highlighting significant upregulated genes in cancer cells of each region. **E**. Comparison of colocalization and cell-cell communication results. Plain line (top axis) represents the count of significant ligand-receptor interactions between each of the defined cancer cells and the y-axis cells. Dotted line (bottom axis) indicates colocalization, measured by the Jaccard similarity index based on the presence or absence of cells within each spot. **F**. Spearman correlation between colocalization and ligand-receptor counts. Color code: violet cancer cells in location 1, orange cancer cells in location 2.

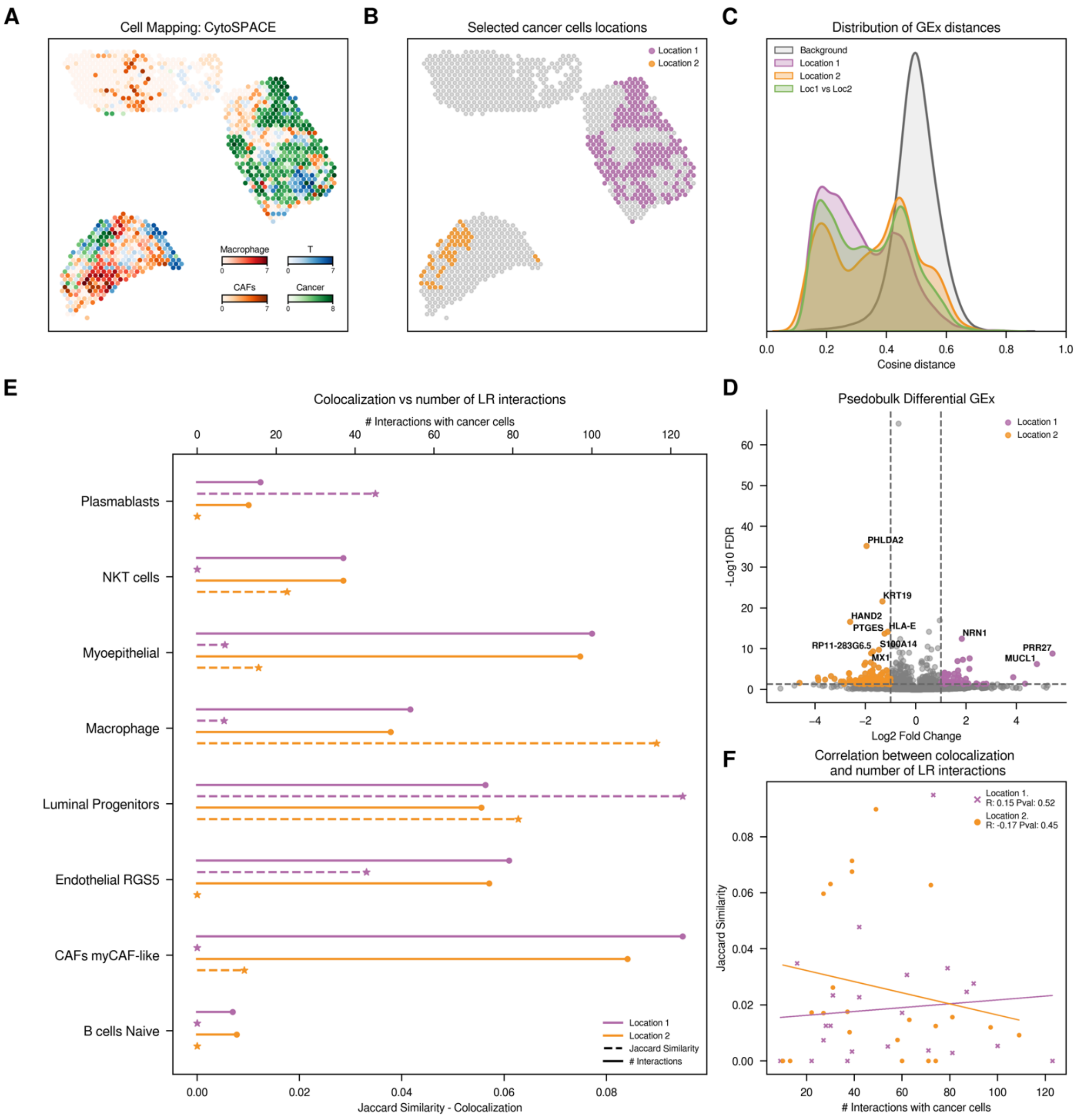

Fig S5. Exploration of cancer cells from CID44971 patient. A. Distribution of main cell types across the tissue slide. For visualization purposes, we normalized the CytoSPACE inferred abundances of predominant cell types, with each spatial spot representing the cell type of highest abundance. **B.** Spatial representation of cancer cells according to their assigned location. **C.** Distribution of cosine distances between single-cell gene expression (GEx) profiles. We analyze the cosine distances between gene expression patterns of cancer cells located within the same or different tissue regions. **D.** Volcano plot with Differential Gene Expression results, highlighting significant upregulated genes in cancer cells of each region. **E.** Comparison of colocalization and CellPhoneDB cell-cell communication results. Plain line (top axis) represents the count of significant ligand-receptor interactions between each of the defined cancer cells and the y-axis cells. Dotted line (bottom axis) indicates colocalization, measured by the Jaccard similarity index based on the presence or absence of cells within each spot. **F.** Spearman correlation between colocalization and ligand-receptor interaction counts. Color code: Violet cancer cells in location 1, orange cancer cells in location 2.

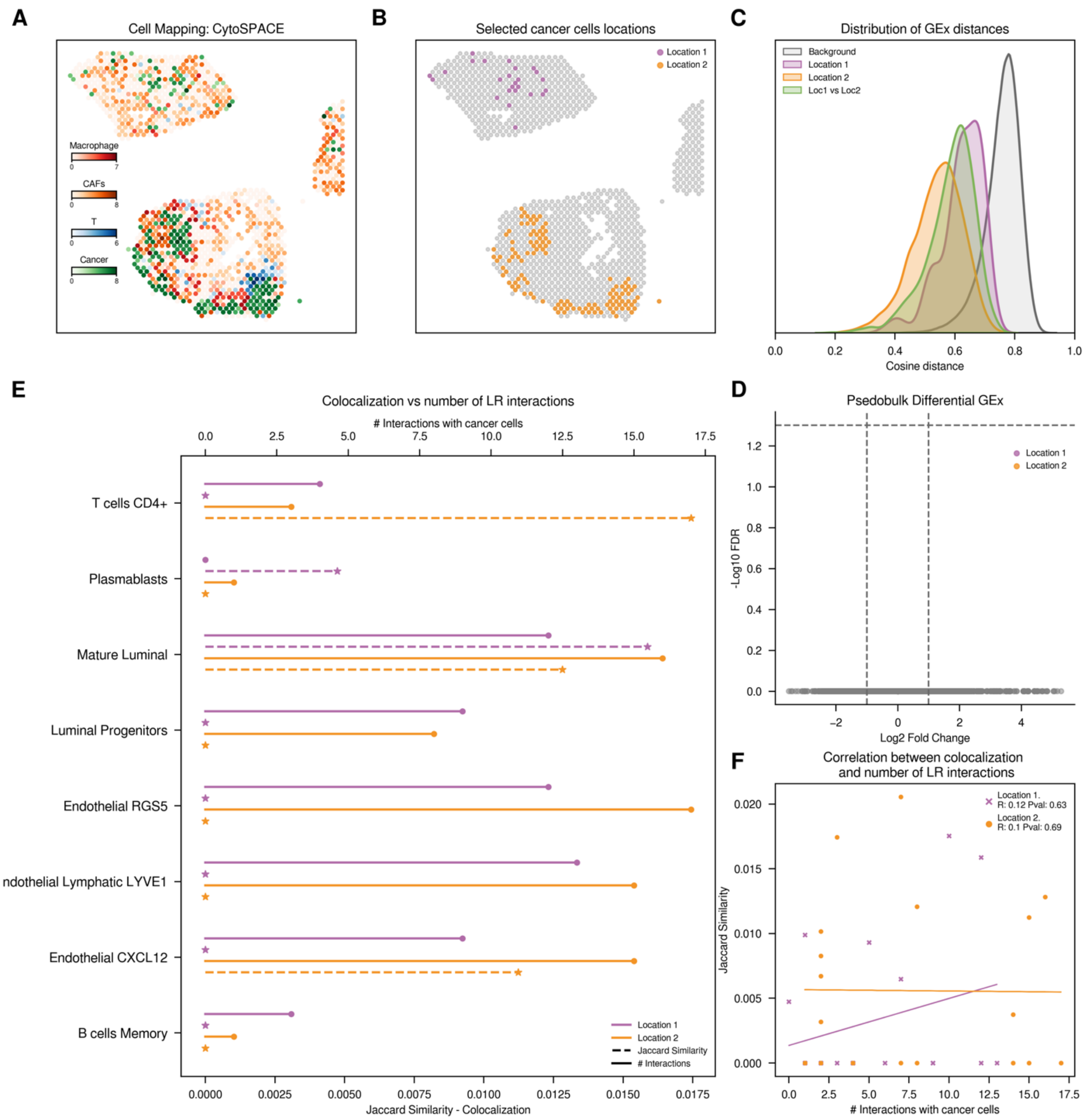

Fig S6. Exploration of cancer cells from CID4465 patient. A. Distribution of main cell types across the tissue slide. For visualization purposes, we normalized the CytoSPACE inferred abundances of predominant cell types, with each spatial spot representing the cell type of highest abundance. **B.** Spatial representation of cancer cells according to their assigned location. **C.** Distribution of cosine distances between single-cell gene expression (GEx) profiles. We analyze the cosine distances between gene expression patterns of cancer cells located within the same or different tissue regions. **D.** Volcano plot with Differential Gene Expression results, highlighting no significant upregulated genes in cancer cells of each region. **E.** Comparison of colocalization and CellPhoneDB cell-cell communication results. Plain line (top axis) represents the count of significant ligand-receptor interactions between each of the defined cancer cells and the y-axis cells. Dotted line (bottom axis) indicates colocalization, measured by the Jaccard similarity index based on the presence or absence of cells within each spot. **F.** Spearman correlation between colocalization and ligand-receptor interaction counts. Color code: Violet cancer cells in location 1, orange cancer cells in location 2.

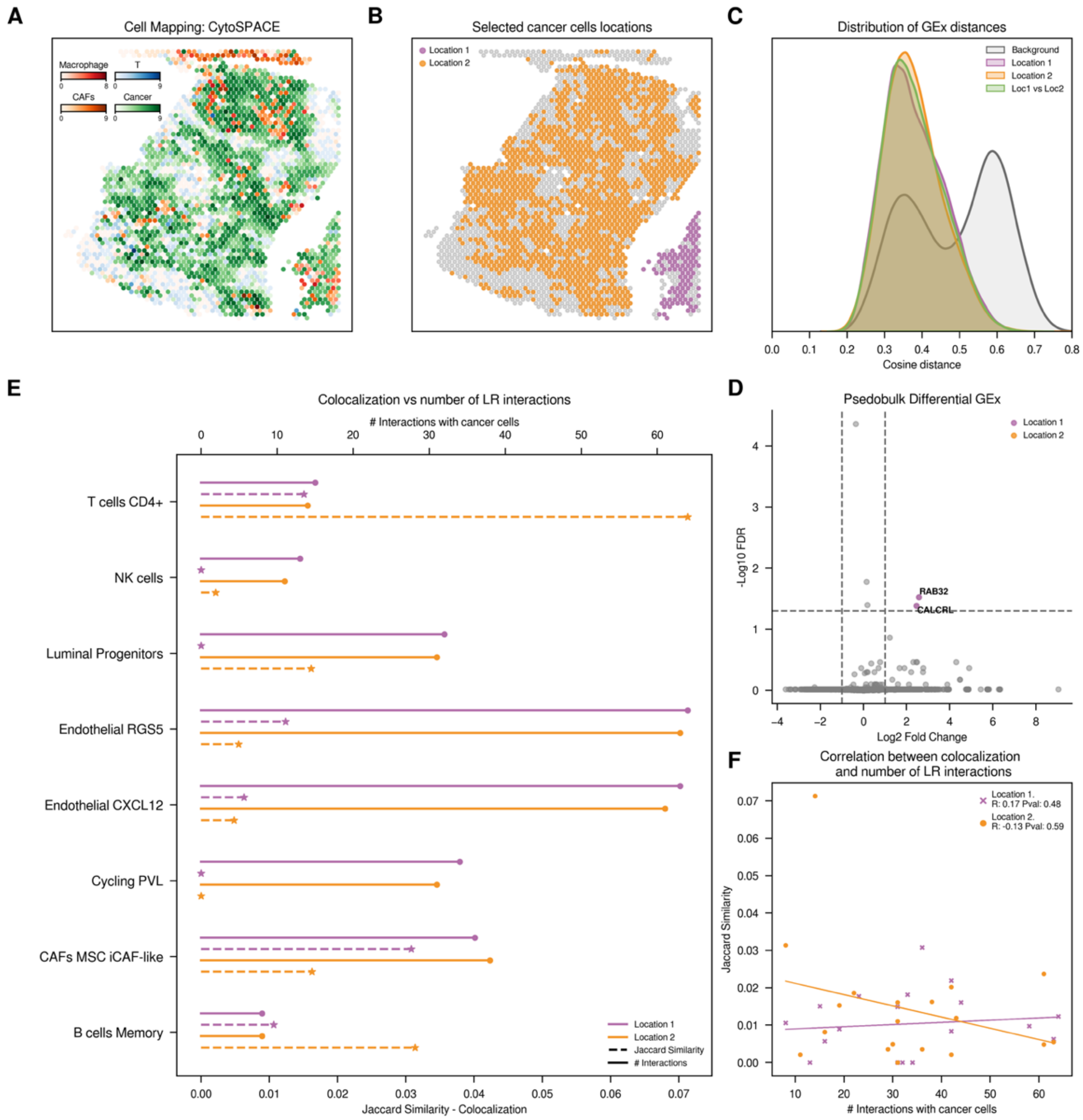

Fig S7. Exploration of cancer cells from CID4290 patient. A. Distribution of main cell types across the tissue slide. For visualization purposes, we normalized the CytoSPACE inferred abundances of predominant cell types, with each spatial spot representing the cell type of highest abundance. **B.** Spatial representation of cancer cells according to their assigned location. **C.** Distribution of cosine distances between single-cell gene expression (GEx) profiles. We analyze the cosine distances between gene expression patterns of cancer cells located within the same or different tissue regions. **D.** Volcano plot with Differential Gene Expression results, highlighting significant upregulated genes in cancer cells of only one region. **E.** Comparison of colocalization and CellPhoneDB cell-cell communication results. Plain line (top axis) represents the count of significant ligand-receptor interactions between each of the defined cancer cells and the y-axis cells. Dotted line (bottom axis) indicates colocalization, measured by the Jaccard similarity index based on the presence or absence of cells within each spot. **F.** Spearman correlation between colocalization and ligand-receptor interaction counts. Color code: Violet cancer cells in location 1, orange cancer cells in location 2.

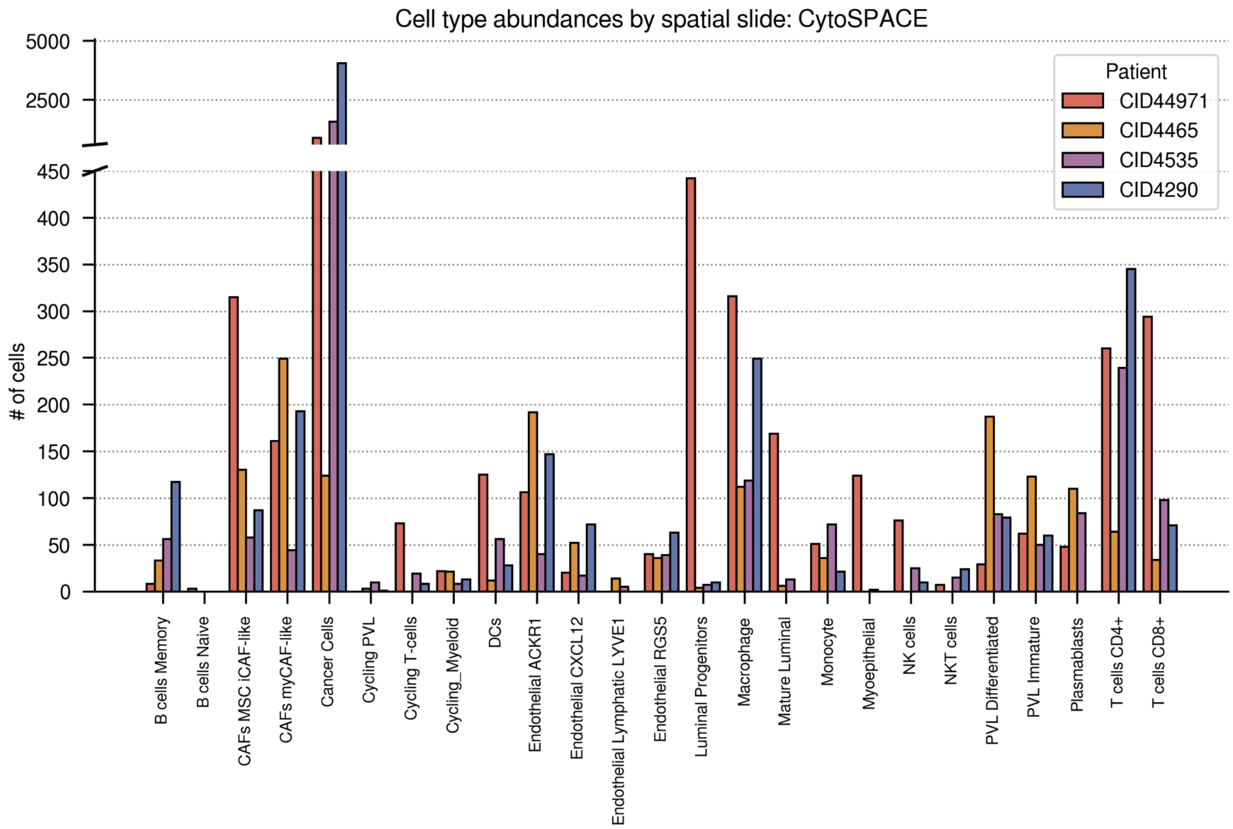

Fig S8. **Variability of cell type abundance across breast cancer patient tissue samples.** Bar plot showing the distribution of cell types, determined using CytoSPACE, across spatial slides from the four breast cancer patients. Abundances were calculated by summing the number of cells of each type mapped to individual spots within the CytoSPACE results. Each bar represents the count of a specific cell type within a slide, and distinct colors are assigned to each patient to facilitate comparison.

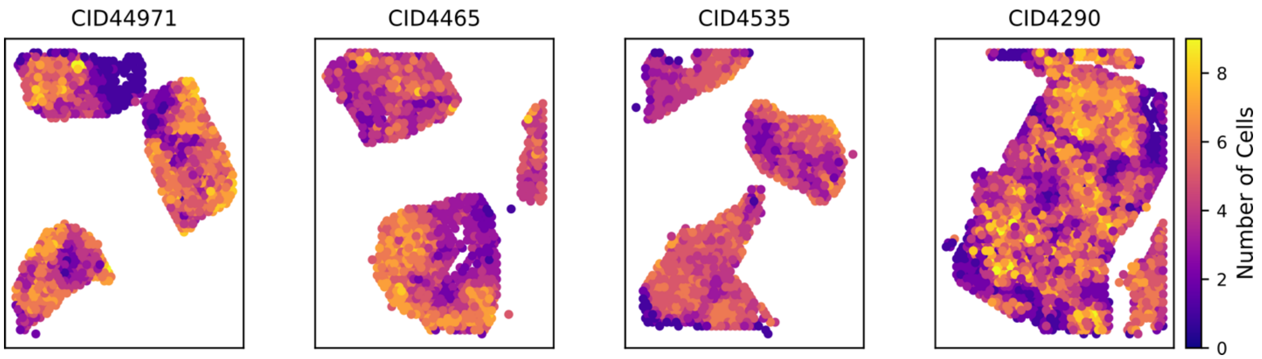

Fig S9. Estimated number of cells per spatial spot using CytoSPACE in the Brest Cancer patient samples. Visualization of cellular density across the analyzed tissue sections. Color gradient represents the estimated number of cells mapped per spatial spot.

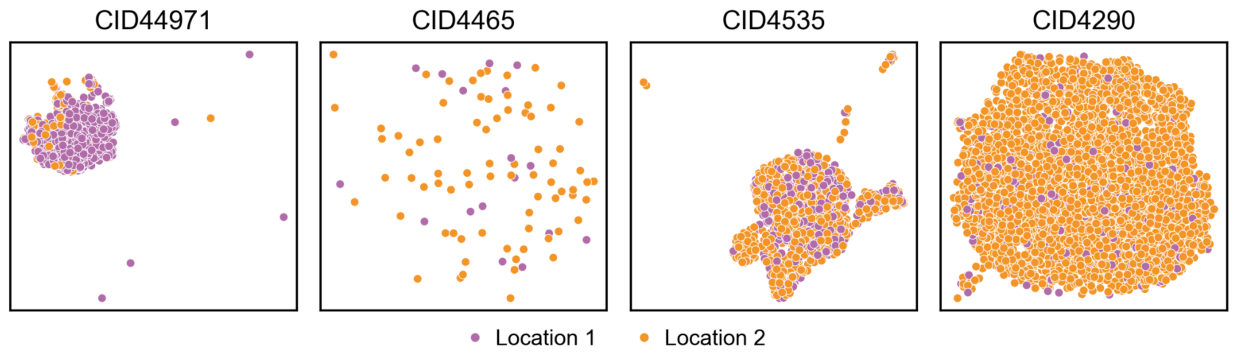

Fig S10. **UMAP projection of cancer cell gene expression by location.** Two-dimensional Uniform Manifold Approximation and Projection (UMAP) visualization of the single-cell gene expression profiles of cancer cells by patient, to explore the potential unsupervised separation based on the assigned location. Each point represents an individual cancer cell, colored according to its assigned location.

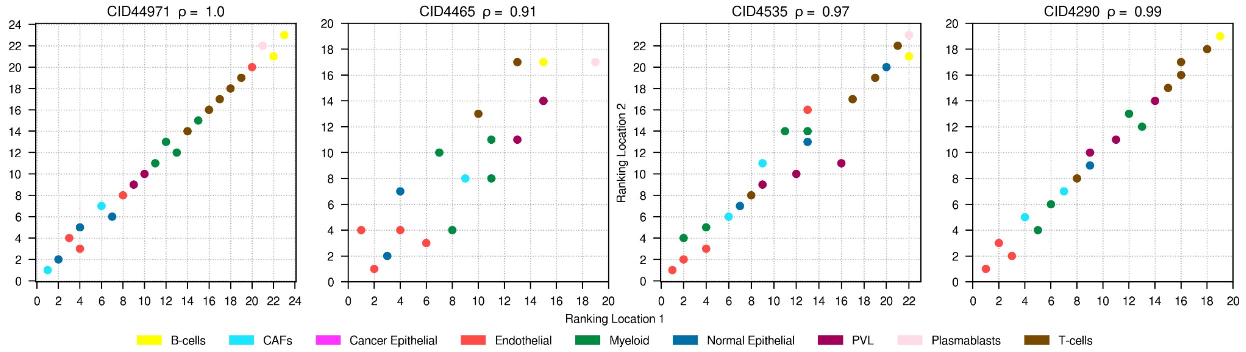

Fig S11. **Correlation of interaction frequency between non-cancer cells and cancer cells across different tumor regions.** Using the interactions inferred by CellPhoneDB, we analyzed the correlation in interaction frequency between non-cancer cells and cancer cells in each of the defined regions. Spearman correlation coefficients - 0.995, 0.91, 0.97, 0.99- reveal a strong correlation in interaction frequency across both groups of spatially defined cancer cells for all samples.

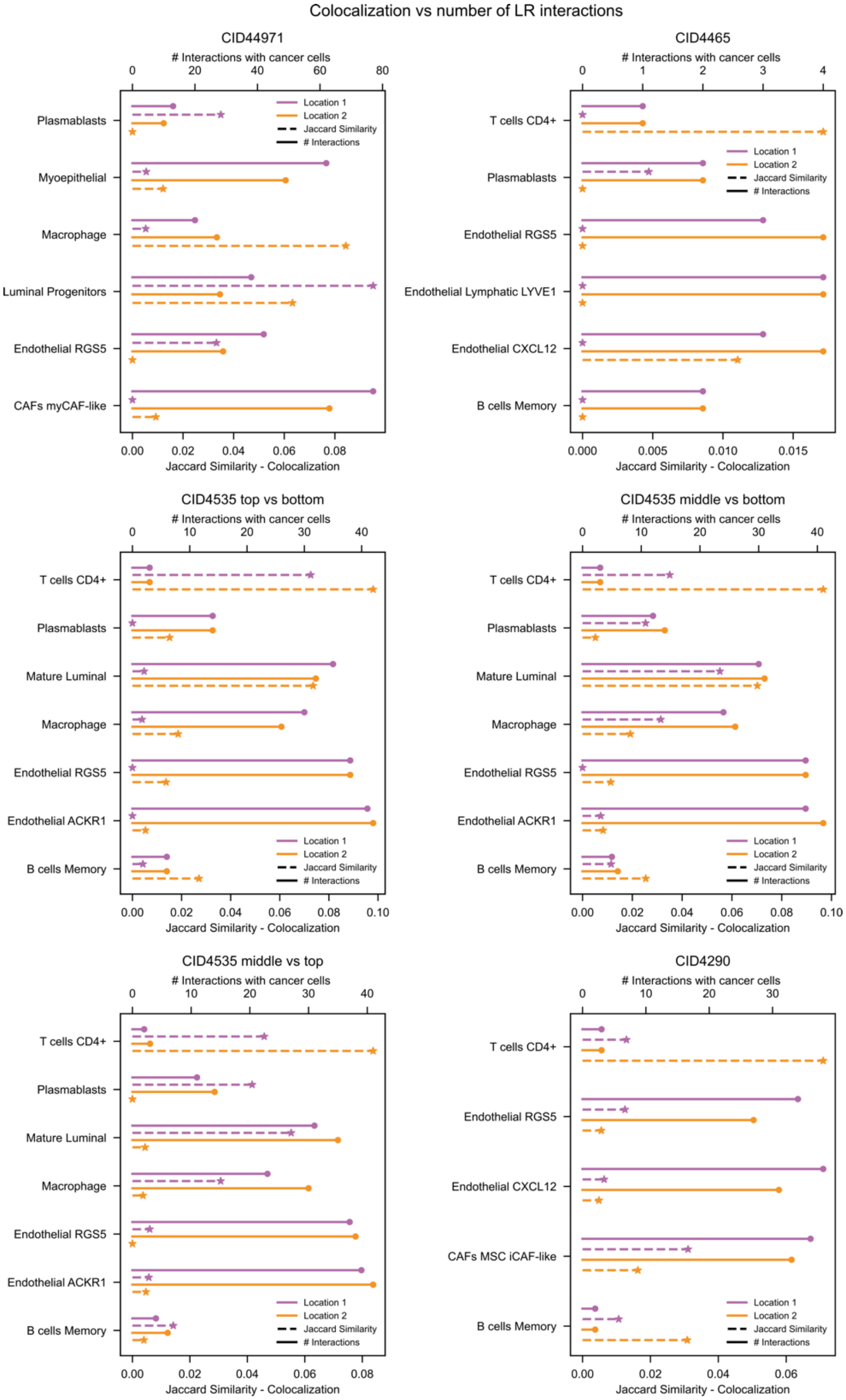

Fig S12. Comparison between colocalization and CellChat cell-cell communication results across four breast cancer samples. This figure presents a comparative analysis of colocalization results and the number of ligand-receptor interactions predicted between location-defined cancer cells and cells in the y-axis across four breast cancer samples. Plain line represents the number of significant ligand-receptor interactions identified by CellChat. Dotted line illustrates the degree of colocalization, measured as the Jaccard similarity index between the spot profiles of cancer cells and y-axis cells (based on the presence or absence of cells in each spot). For visualization purposes, we selected the extreme cases, those with the highest and lowest levels of colocalization and interaction frequencies.

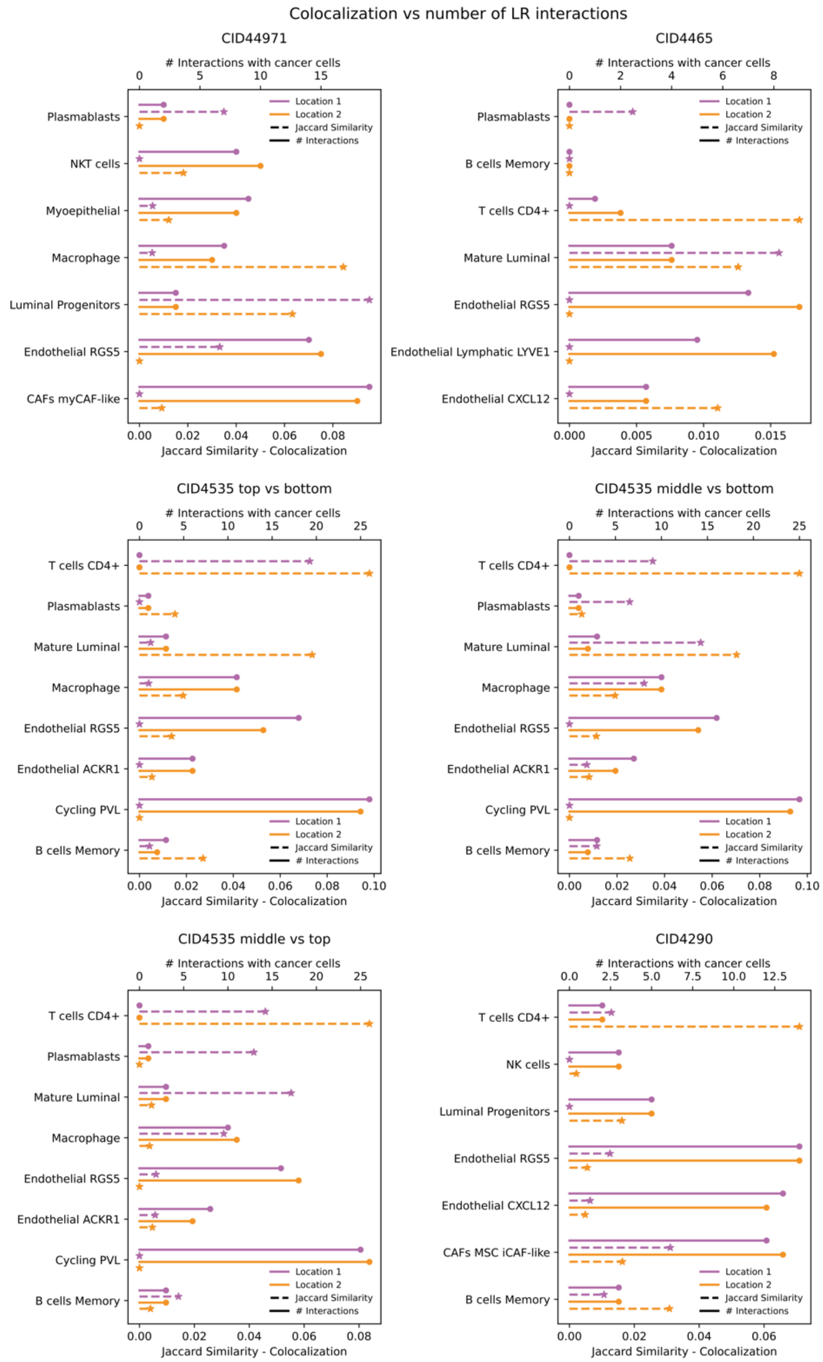

Fig S13. Comparison of colocalization and LIANA cell-cell communication results across four breast cancer samples. This figure presents a comparative analysis of colocalization results and the number of ligand-receptor interactions predicted between location-defined cancer cells and cells in the y-axis across four breast cancer samples. Plain line represents the number of significant ligand-receptor interactions identified by LIANA, utilizing the consensus score. Dotted line illustrates the degree of colocalization, measured as the Jaccard similarity index between the spot profiles of cancer cells and y-axis cells (based on the presence or absence of cells in each spot). For visualization purposes, we selected the extreme cases, those with the highest and lowest levels of colocalization and interaction frequencies.

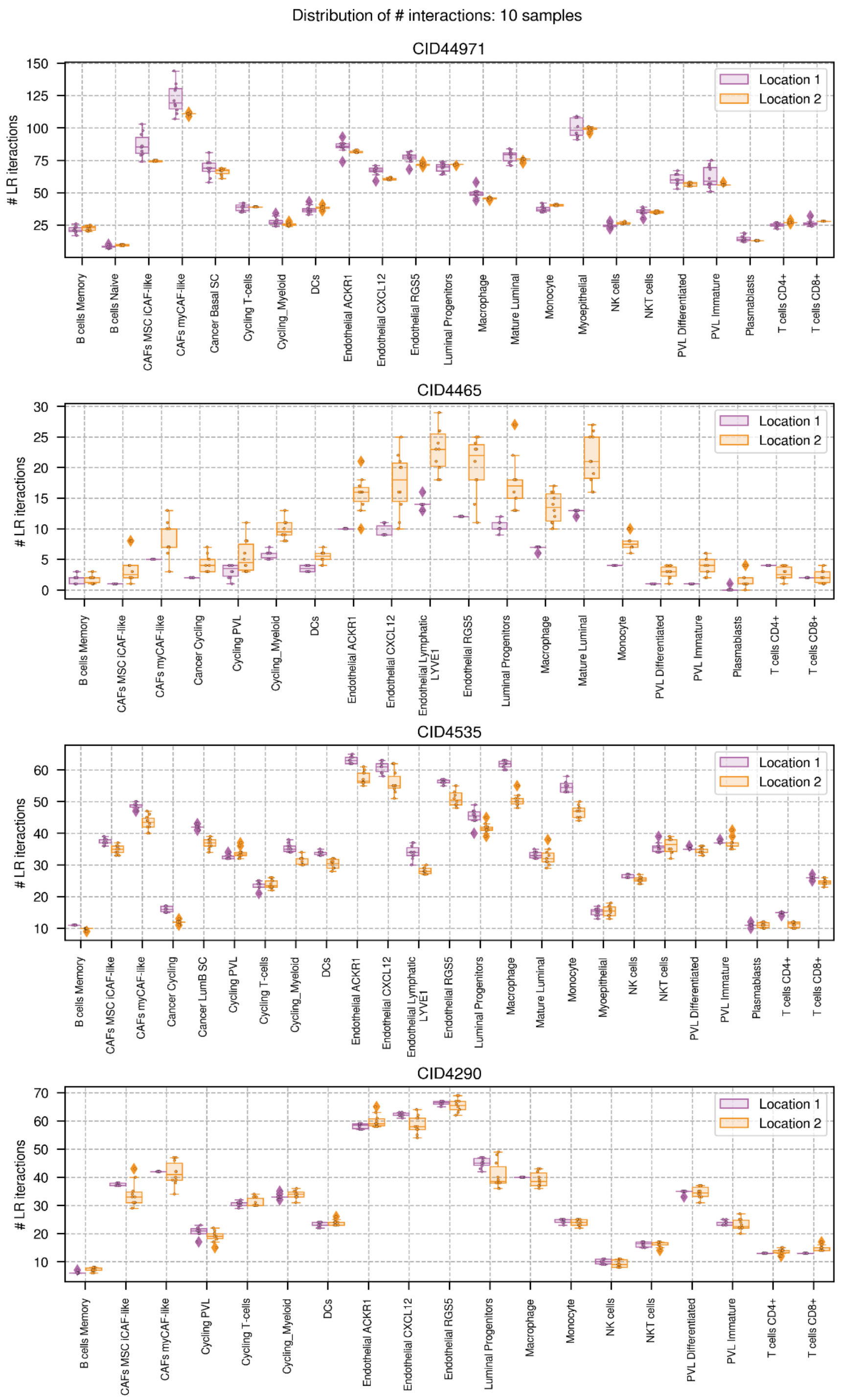

Fig S14. Subsampling exercise in the four breast cancer samples. This figure illustrates the minimal variability in predicted ligand-receptor interactions counts between cancer cells located in different regions and the rest of the cells in the dataset, over several subsampling iterations. To ensure a balanced analysis and mitigate biases due to varying cancer cell quantities in each region, ten unique subsampled datasets were created. Then, we predict the ligand-receptor interactions with CellPhoneDB adjusted for these quantity discrepancies.

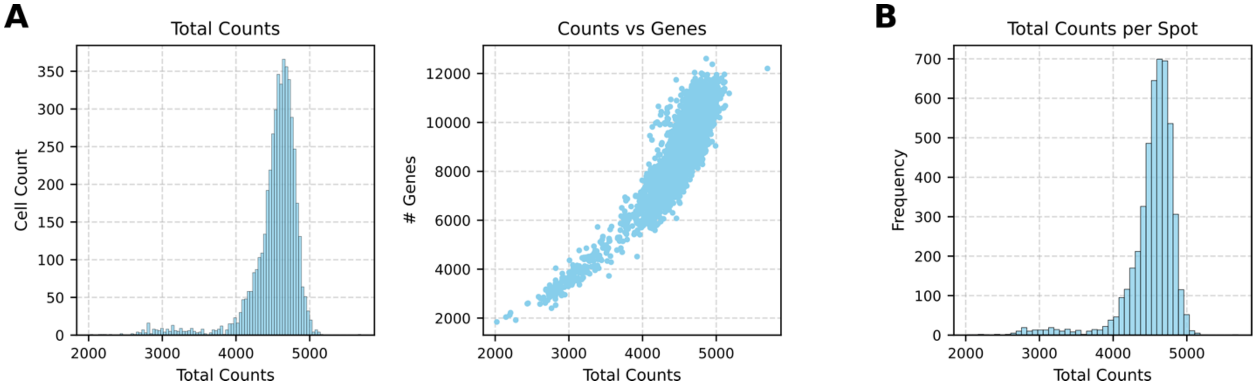

Fig S15. Quality control (QC) metrics for scRNA-seq and spatial transcriptomics data from the Brain dataset. **A**. QC results for brain dataset scRNA-seq data. We represent the distribution of total UMIs (counts per cell) and a scatterplot to illustrate the relationship between total UMIs (x-axis) and detected genes per cell (y-axis). **B**. Distribution of total UMIs per spatial spot for the same patients.

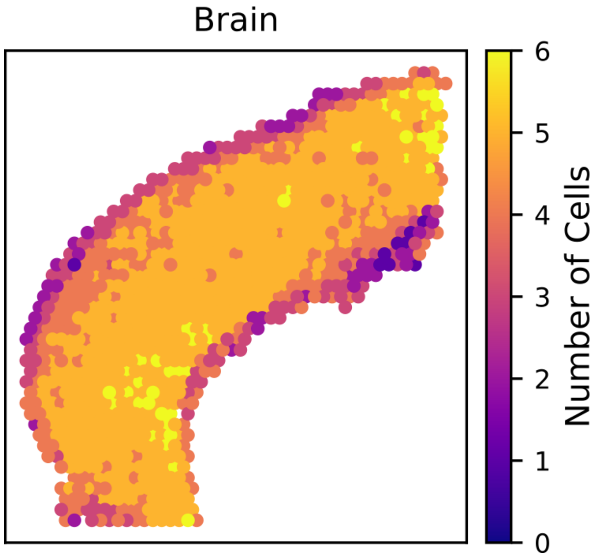

Fig S16. Estimated number of cells per spatial spot using CytoSPACE in the mouse brain sample. Visualization of cellular density across the analyzed tissue sections. Color gradient represents the estimated number of cells mapped per spatial spot.

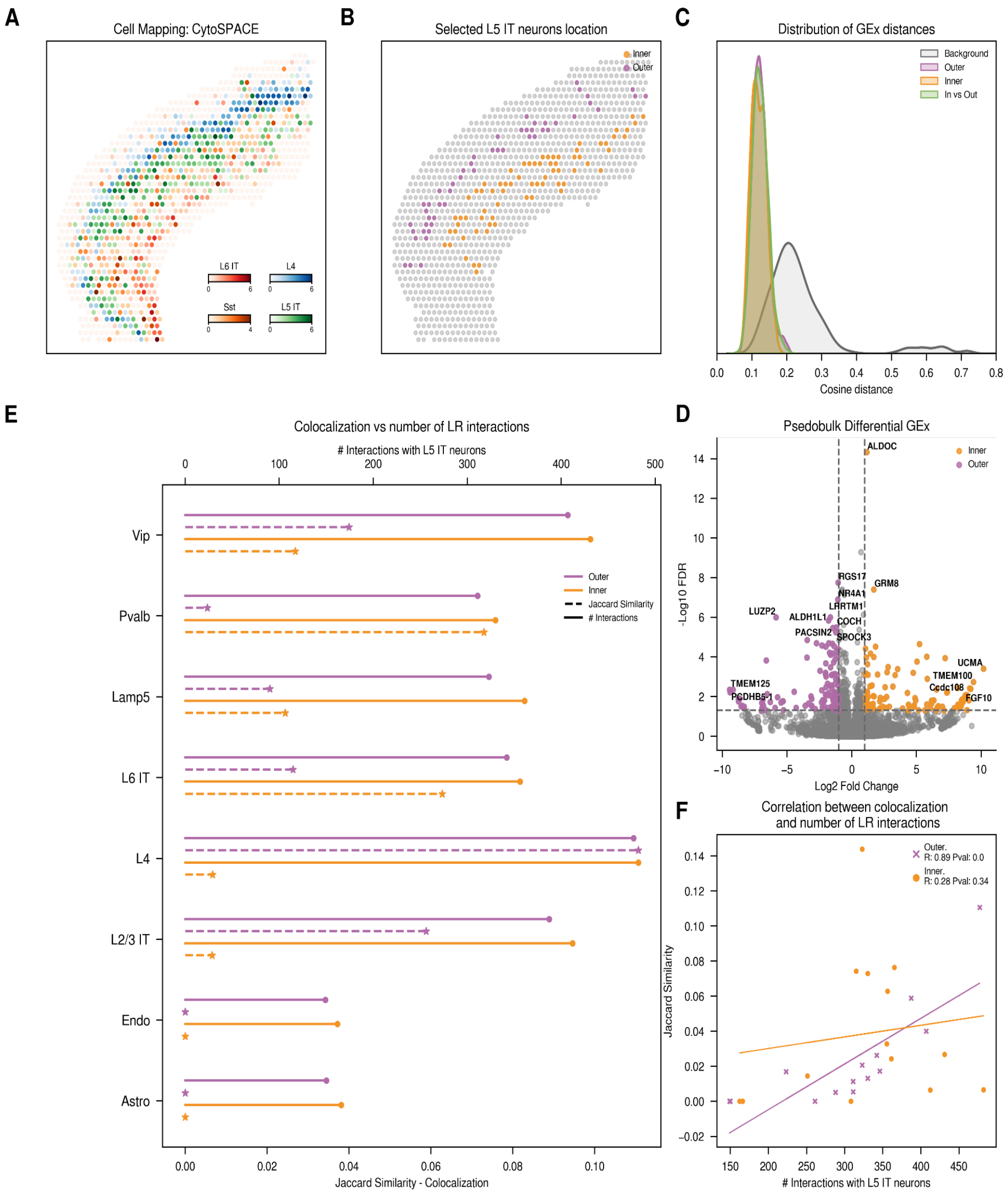

Fig S17. Exploration of L5 IT neurons from mouse brain dataset. **A**. Distribution of adjacent cell types and different neuron types across the tissue slide. For visualization purposes, we normalized the CytoSPACE inferred abundances of selected cell types, with each spatial spot representing the cell type of highest abundance. **B**. Spatial representation of L5 IT neurons according to their assigned location. **C**. Distribution of cosine distances between single-cell gene expression (GEx) profiles. We analyze the cosine distances between gene expression patterns of L5 IT neurons located within the same or different tissue regions (outer or inner part of the L5 layer). **D**. Volcano plot with Differential Gene Expression results, highlighting significant upregulated genes in L5 IT neurons of each region. **E**. Comparison of colocalization and CellPhoneDB cell-cell communication results. Plain line (top axis) represents the count of significant ligand-receptor interactions between each of the defined L5 IT neurons and the y-axis cells. Dotted line (bottom axis) indicates colocalization, measured by the Jaccard similarity index based on the presence or absence of cells within each spot. **F**. Spearman correlation between colocalization and ligand-receptor counts. Color code: Violet neurons in the outer part of the layer, orange neurons in the inner part of the layer.

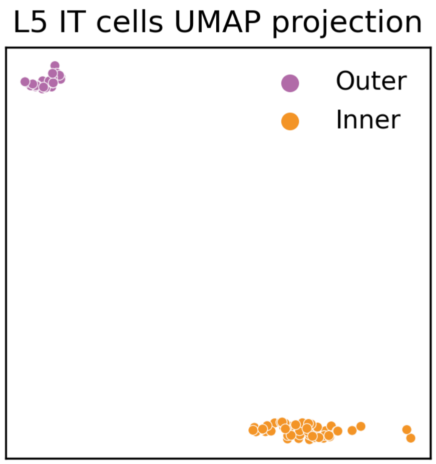

Fig S18. **UMAP projection of L5 IT neurons gene expression by location.** Two-dimensional Uniform Manifold Approximation and Projection (UMAP) visualization of the gene expression profiles of L5 IT neurons, to explore the potential unsupervised separation based on assigned location within the L5 layer. Each point represents an individual neuron, colored according to its assigned location. In this case, the UMAP visualization demonstrates a clear separation between the gene expression profiles of L5 IT neurons positioned in the inner versus the outer neurons.

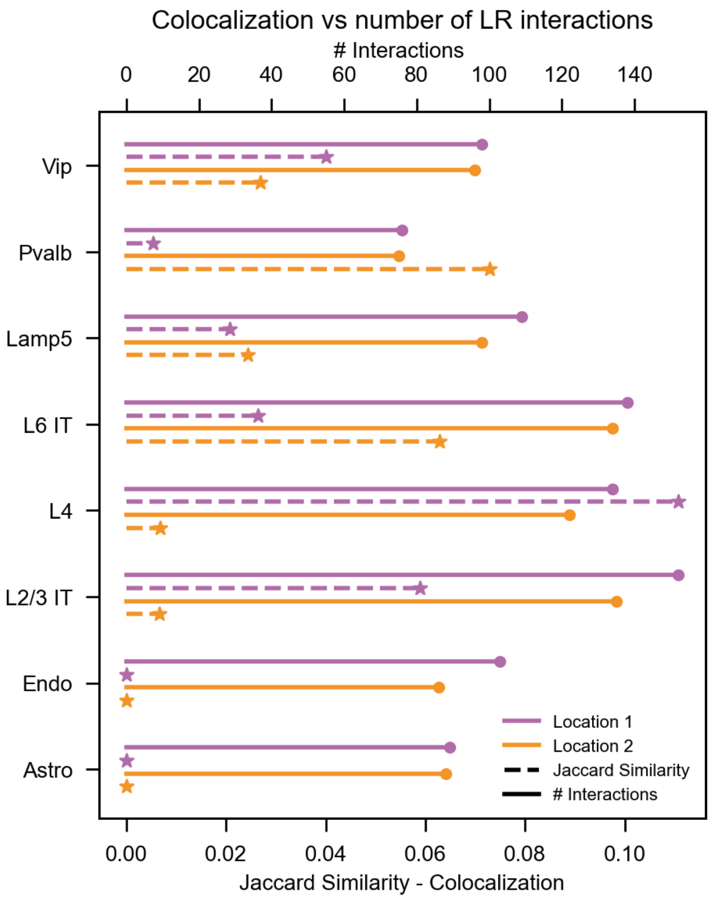

Fig S19. Comparison between colocalization and CellChat Mouse cell-cell communication results in the brain dataset. This figure presents a comparative analysis of colocalization results and the number of ligand-receptor interactions predicted between inner/outer L5 neurons and cells in the y-axis. Plain line represents the number of significant ligand-receptor interactions identified by CellChat, utilizing the consensus score. Dotted line illustrates the degree of colocalization, measured as the Jaccard similarity index between the spot profiles of L5 neurons and y-axis cells (based on the presence or absence of cells in each spot). For visualization purposes, we selected the extreme cases, those with the highest and lowest levels of colocalization and interaction frequencies.

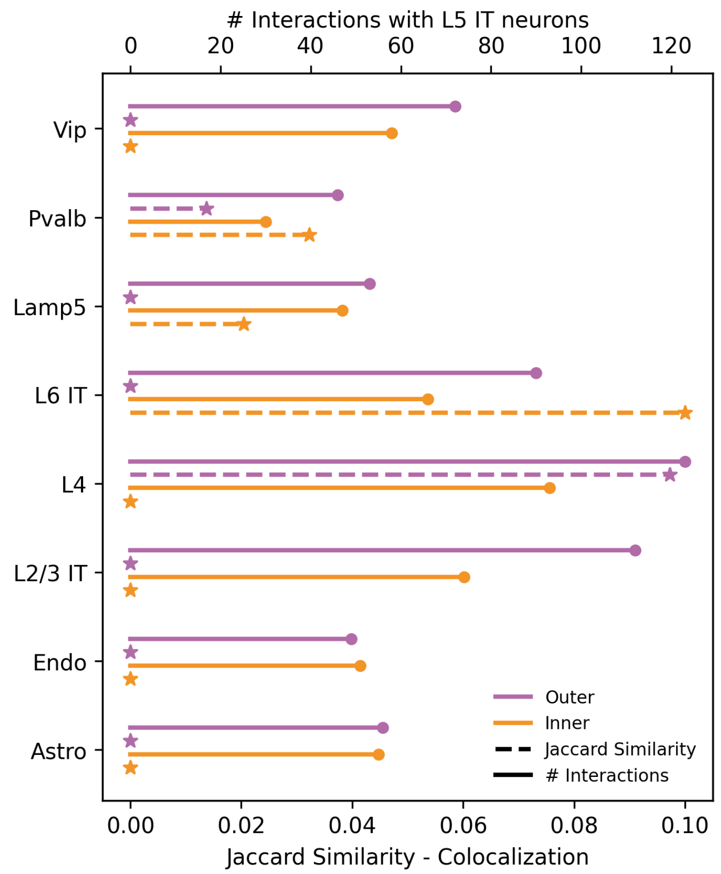

Fig S20. Comparison between colocalization and LIANA cell-cell communication results in the brain dataset. This figure presents a comparative analysis of colocalization results and the number of ligand-receptor interactions predicted between inner/outer L5 neurons and cells in the y-axis. Plain line represents the number of significant ligand-receptor interactions identified by LIANA, utilizing the consensus score. Dotted line illustrates the degree of colocalization, measured as the Jaccard similarity index between the spot profiles of L5 neurons and y-axis cells (based on the presence or absence of cells in each spot). For visualization purposes, we selected the extreme cases, those with the highest and lowest levels of colocalization and interaction frequencies.

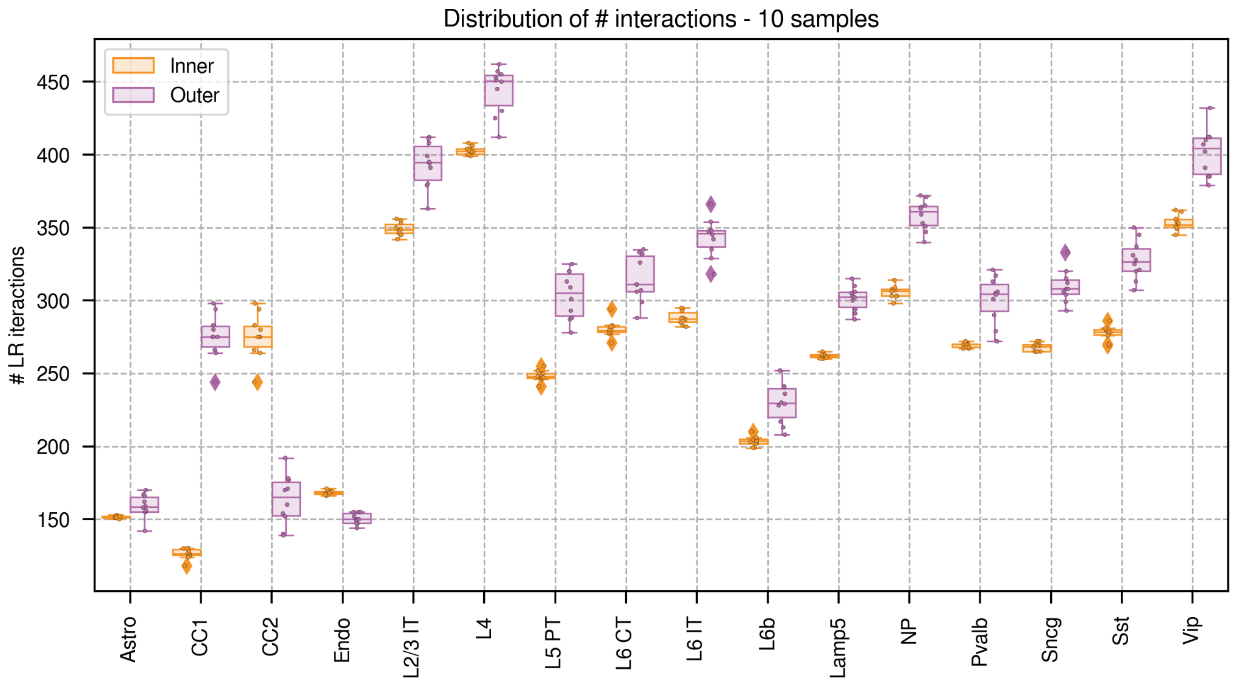

Fig S21. Subsampling exercise. This figure illustrates the minimal variability in predicted ligand-receptor interactions counts between L5 IT neurons located in different parts of the L5 layer and the rest of the cells in the dataset, over several subsampling iterations. To ensure a balanced analysis and mitigate biases due to varying neuron quantities in each region, ten unique subsampled datasets were created. Then, we predict the ligand-receptor interactions with CellPhoneDB adjusted for these quantity discrepancies.

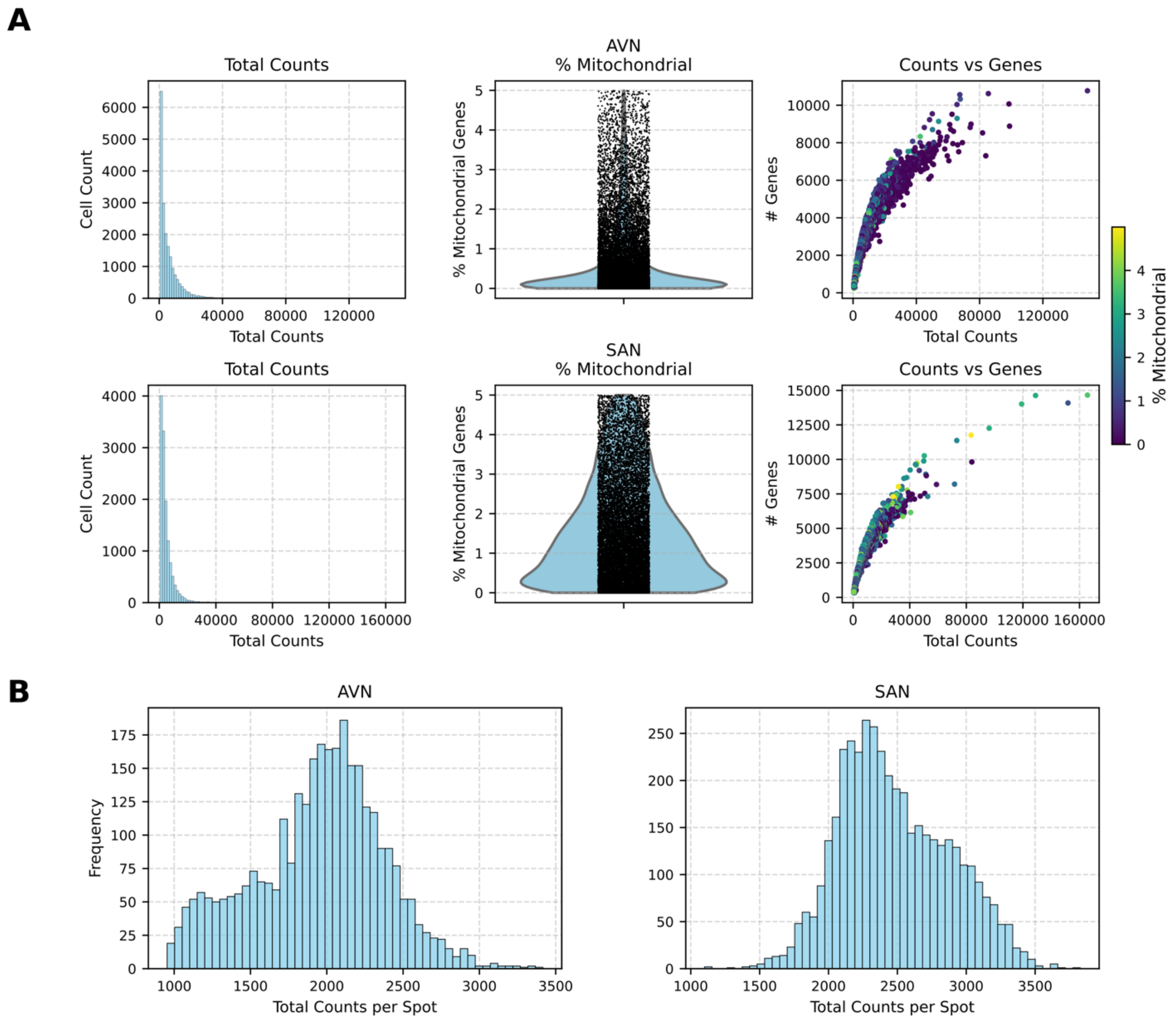

Fig S22. Quality control (QC) metrics for scRNA-seq and spatial transcriptomics data from the Heart dataset by section. **A**. QC results for scRNA-seq data for AVN and SAN regions. For each region, we represent the distribution of total UMIs (counts per cell), a violin plot showing the percentage of mitochondrial gene content and a scatterplot to illustrate the relationship between total UMIs (x-axis) and detected genes per cell (y-axis), colored by mitochondrial gene percentage. **B**. Distribution of total UMIs per spatial spot for the same regions.

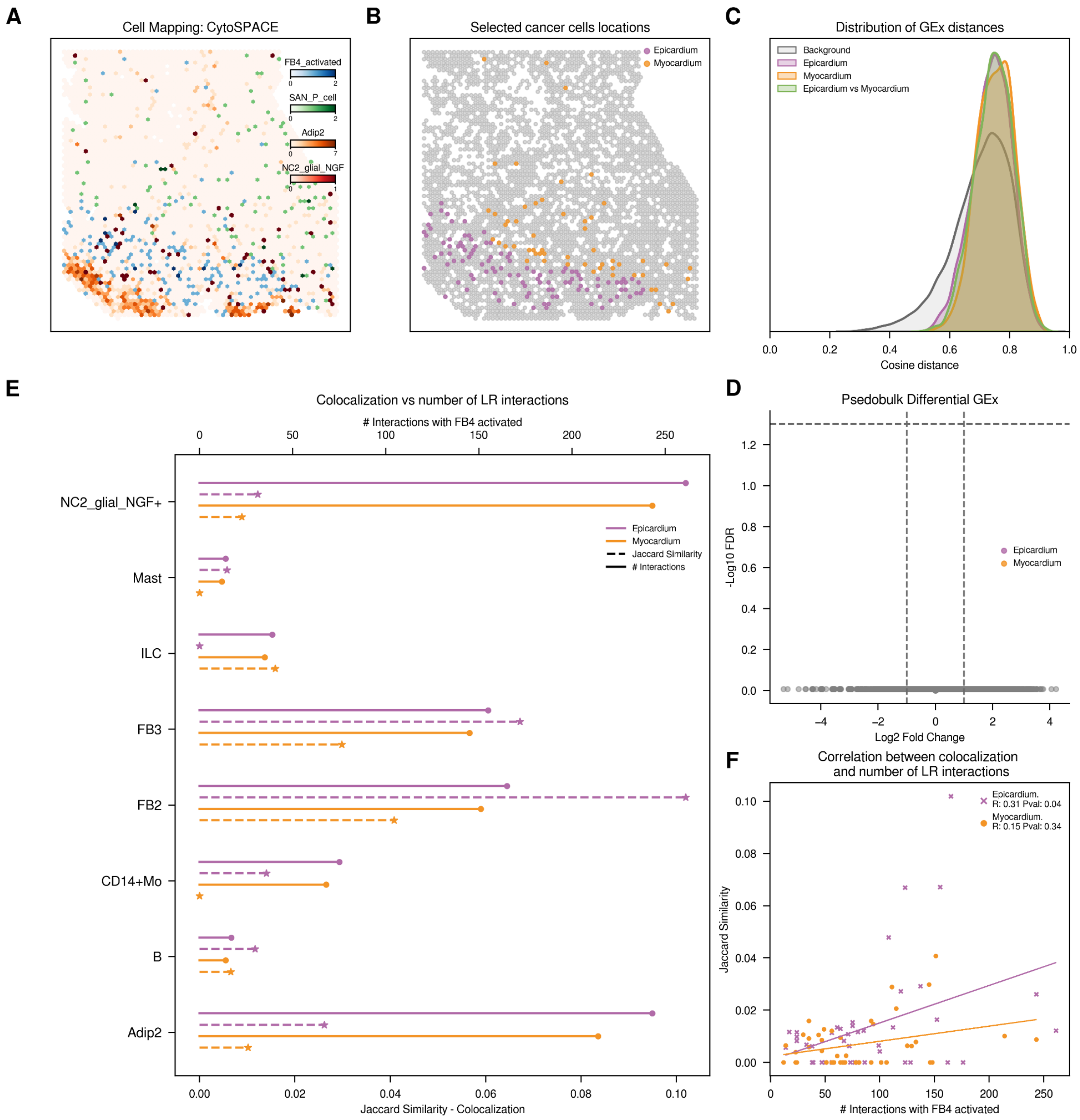

Fig S23. Exploration of fibroblast (FB4_activated) from SAN slide. A. Distribution of main cell types across the tissue slide. For visualization purposes, we normalized the CytoSPACE inferred abundances of predominant cell types, with each spatial spot representing the cell type of highest abundance. **B**. Spatial representation of fibroblasts according to their assigned location. **C**. Distribution of cosine distances between single-cell gene expression (GEx) profiles. We analyze the cosine distances between gene expression patterns of fibroblasts located within the same or different tissue histological regions. **D**. Volcano plot with Differential Gene Expression results, highlighting no significant upregulated genes in fibroblasts of each histological region. **E**. Comparison of colocalization and CellPhoneDB cell-cell communication results. Plain line (top axis) represents the count of significant ligand-receptor interactions between each of the defined fibroblasts and the y-axis cells. Dotted line (bottom axis) indicates colocalization, measured by the Jaccard similarity index based on the presence or absence of cells within each spot. **F**. Spearman correlation between colocalization and ligand-receptor interaction counts. Color code: Violet fibroblasts in the epicardium, orange fibroblasts in the myocardium.

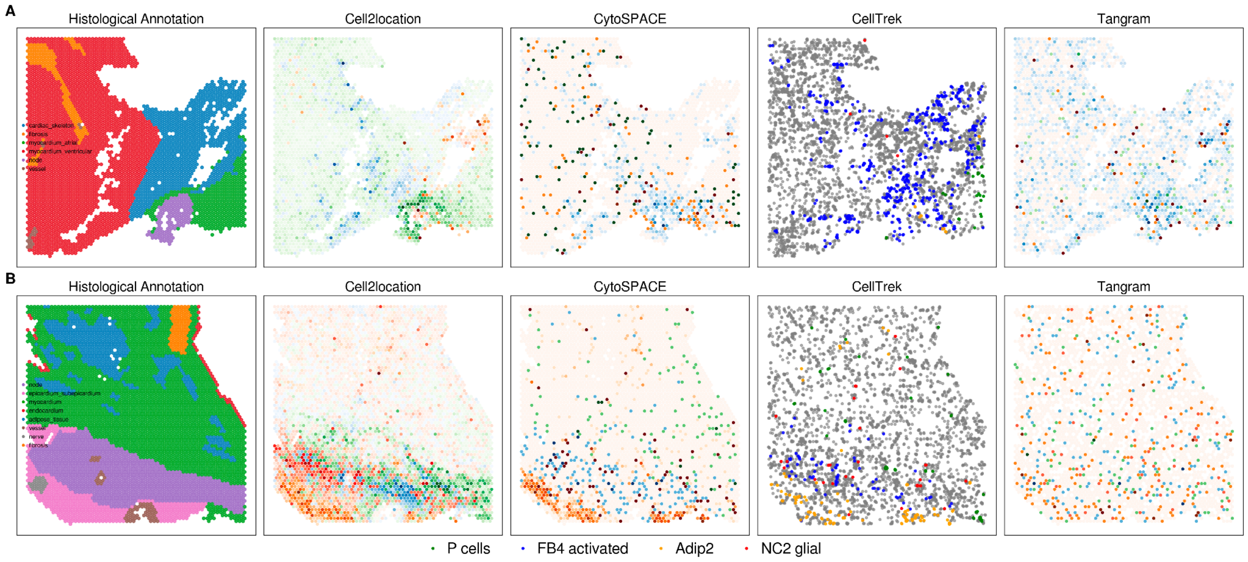

Fig S24. Methodological comparison for disentangling single-cell spatial resolution for Human Heart Atlas dataset. We applied four methodologies - Cell2location, CytoSPACE, CellTrek and Tangram - to determine cell positioning across different heart regions (identified as AVN and SAN, and labeled **A**, **B** respectively). Histological annotations from the original authors are included for comparative analysis (Kanemaru et al., 2023), showing partial agreement between histological and Cell2location results (provided by the authors) and the rest of the cell mapping results. Notably, we were unable to map P cells into the histologically annotated nodes. The figure illustrates the normalized abundance of predominant cell types, with each spatial spot representing the cell type of highest abundance for clear visualization. Color coding: green P cells, blue Fibroblast 4 activated, red NC2 glial and orange Adipcites 2.

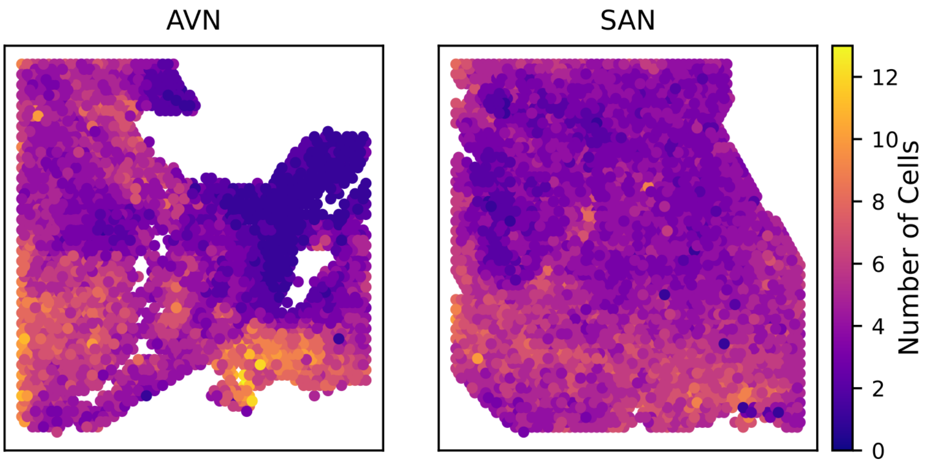

Fig S25. Estimated number of cells per spatial spot using CytoSPACE in Heart samples. Visualization of cellular density across the analyzed tissue sections. Color gradient represents the estimated number of cells mapped per spatial spot.

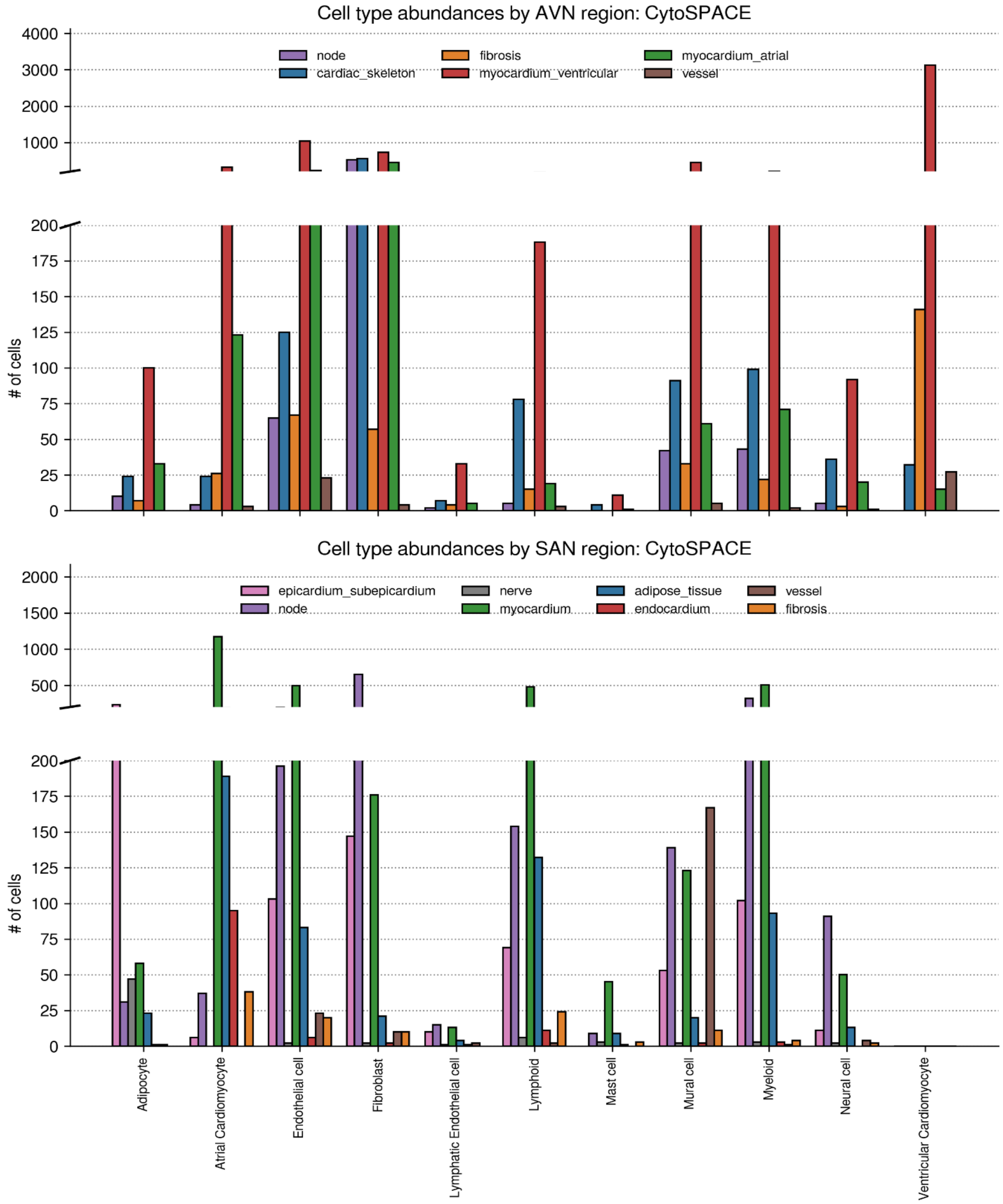

Fig S26. Variability of cell composition across histological regions in AVN and SAN slides. Bar plot illustrating the distribution of cell types, determined using CytoSPACE, across the different histological regions defined within AVN and SAN slides. Abundances were calculated by summing the number of cells of each type mapped to specific spots corresponding to the histological regions within the CytoSPACE results. The figure revealed significant compositional variations based on histological region. For instance, the node region on the SAN slide showed a more diverse cell composition compared to the node region in the AVN slide, which was largely dominated by fibroblasts. Each bar represents the count of a specific cell type within histological regions, and distinct colors are assigned to each histological region to facilitate comparison.

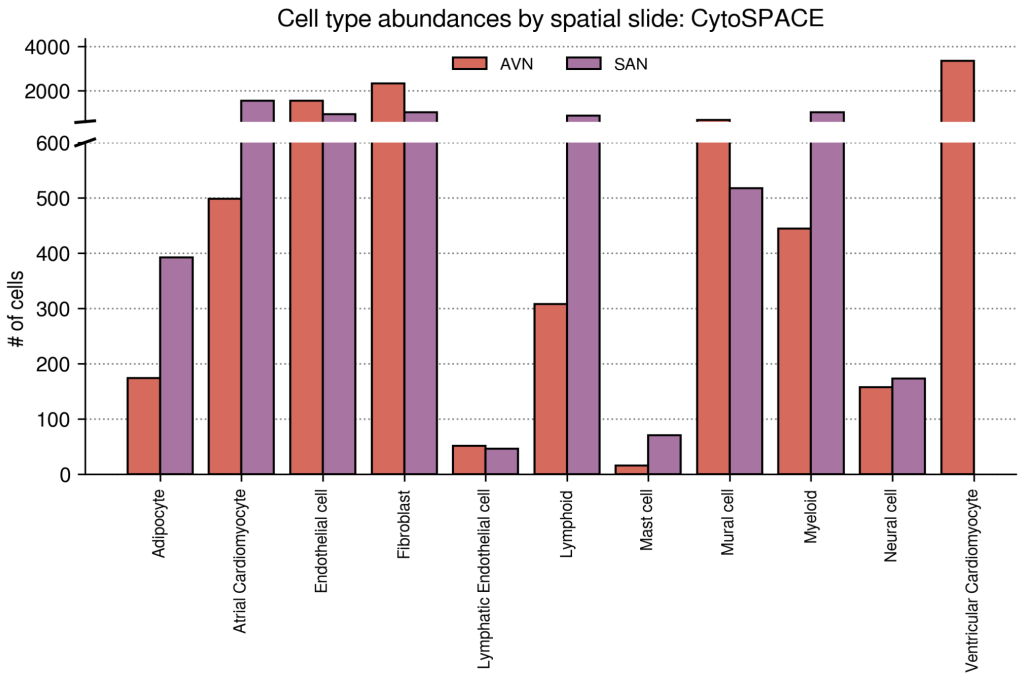

Fig S27. **Variability of cell type abundance across heart regions**. Bar plot showing the distribution of cell types, determined using CytoSPACE, across AVN and SAN slides. Abundances were calculated by summing the number of cells of each type mapped to individual spots within the CytoSPACE results. The figure highlights similar cell type proportions across slides, except for ventricular cardiomyocytes exclusive to the AVN slide and a substantial difference in lymphoid cell presence. Each bar represents the count of a specific cell type within a slide, and distinct colors are assigned to each region to facilitate comparison.

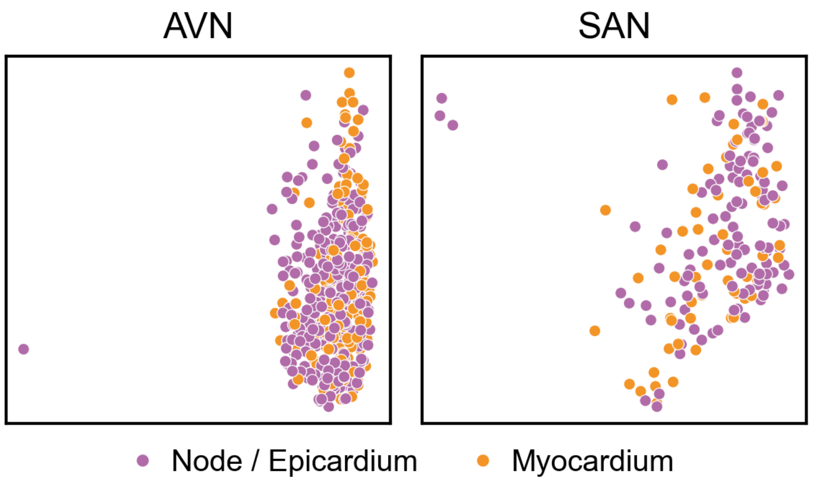

Fig S28. **UMAP projection of fibroblast gene expression by location.** Two-dimensional Uniform Manifold Approximation and Projection (UMAP) visualization of the single-cell gene expression profiles of fibroblast (FB4_activated) in AVN and SAN regions, aimed to explore the potential unsupervised separation based on the fibroblast assigned location. Each point represents an individual fibroblast, colored according to its assigned location. The distribution appears homogeneously across the UMAP space for both slides, suggesting a lack of differences in the gene expression profiles of fibroblast across different histological regions.

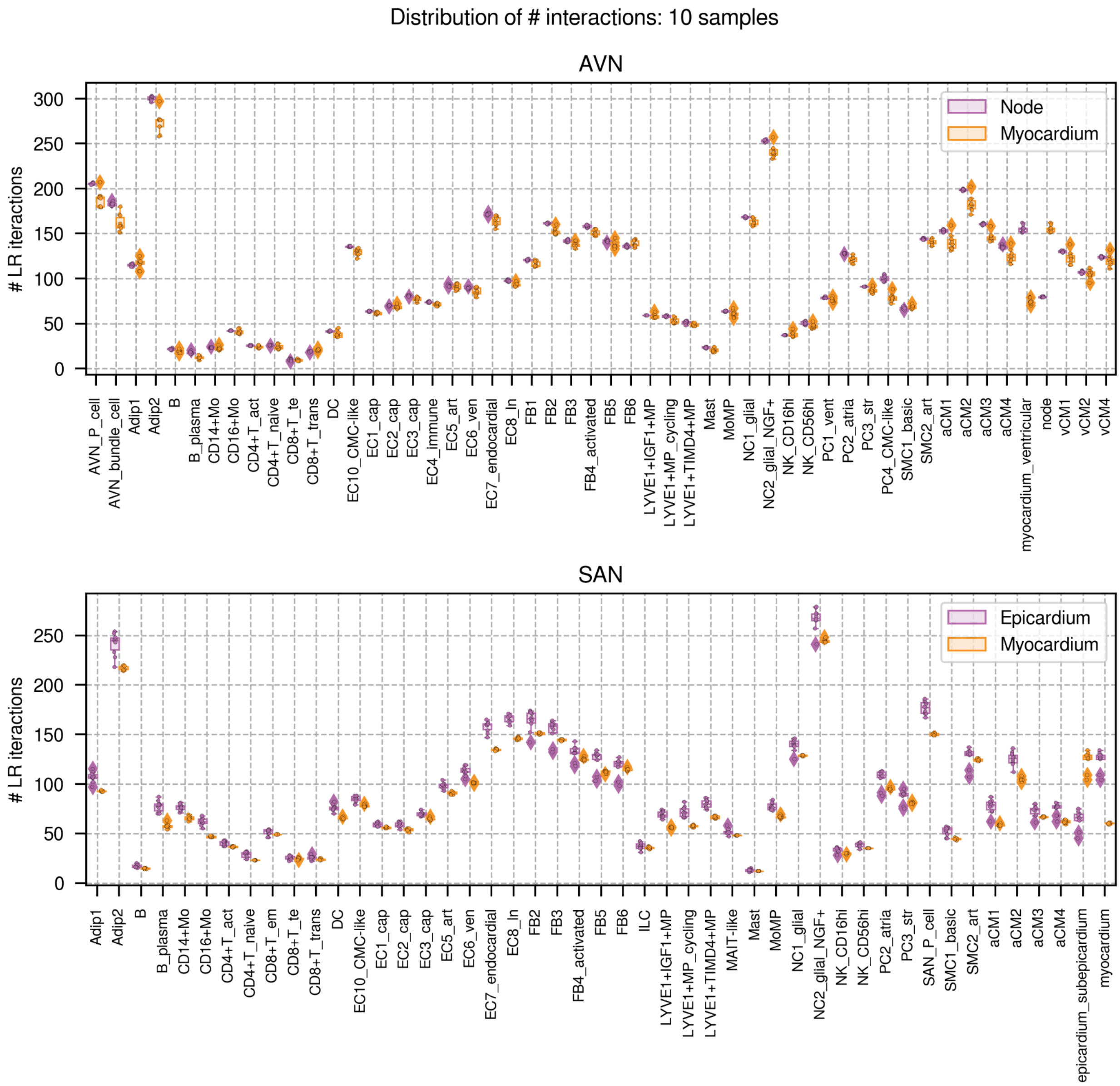

Fig S29. Subsampling exercise in AVN and SAN slides. This figure illustrates the minimal variability in predicted ligand-receptor interactions counts between fibroblasts (FB4_activated) located in different histological regions and the rest of the cells in the dataset, over several subsampling iterations. To ensure a balanced analysis and mitigate biases due to varying fibroblast quantities in each region, ten unique subsampled datasets were created. Then, we predict the ligand-receptor interactions with CellPhoneDB adjusted for these quantity discrepancies.

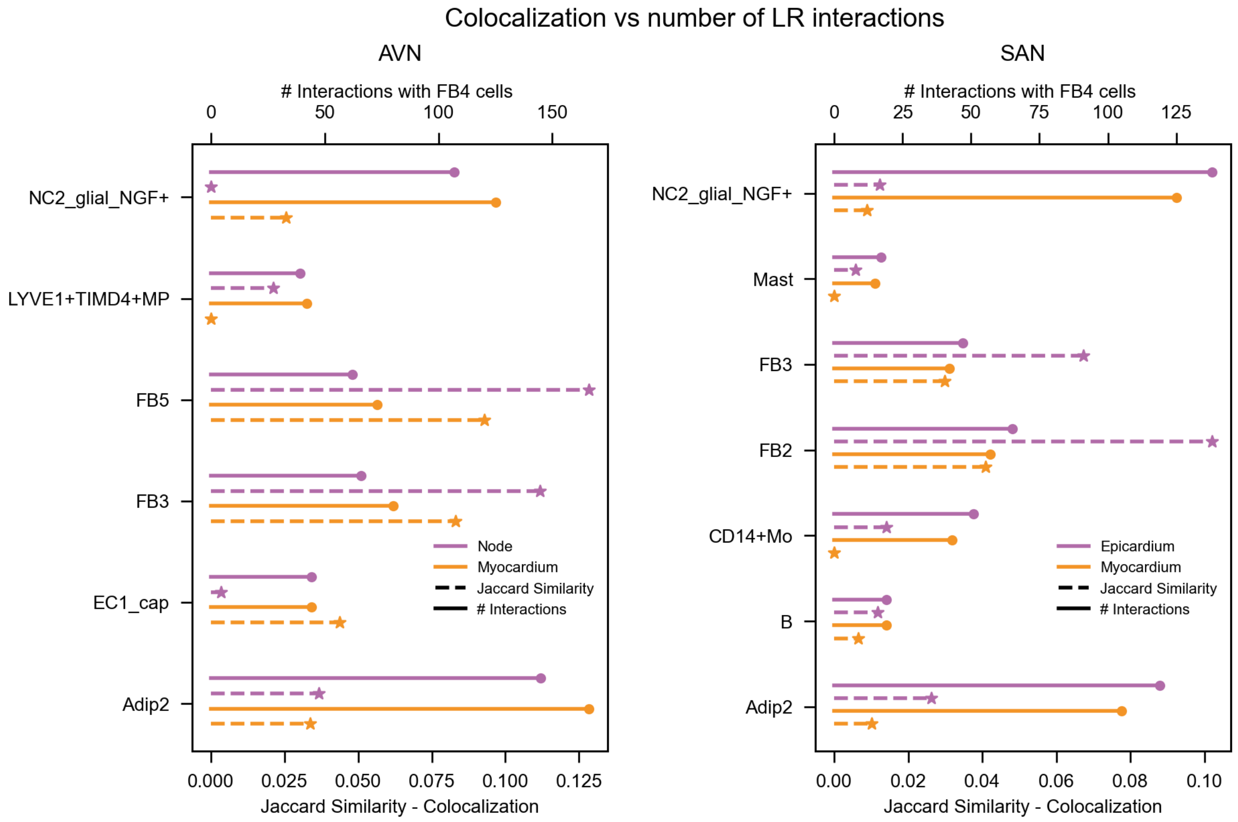

Fig S30. Comparison between colocalization and CellChat cell-cell communication results in AVN and SAN slides. This figure presents a comparative analysis of colocalization results and the number of ligand-receptor interactions predicted between histologically-defined fibroblasts and cells in the y-axis. Plain line represents the number of significant ligand-receptor interactions identified by CellChat, utilizing the consensus score. Dotted line illustrates the degree of colocalization, measured as the Jaccard similarity index between the spot profiles of fibroblasts and y-axis cells (based on the presence or absence of cells in each spot). For visualization purposes, we selected the extreme cases, those with the highest and lowest levels of colocalization and interaction frequencies.

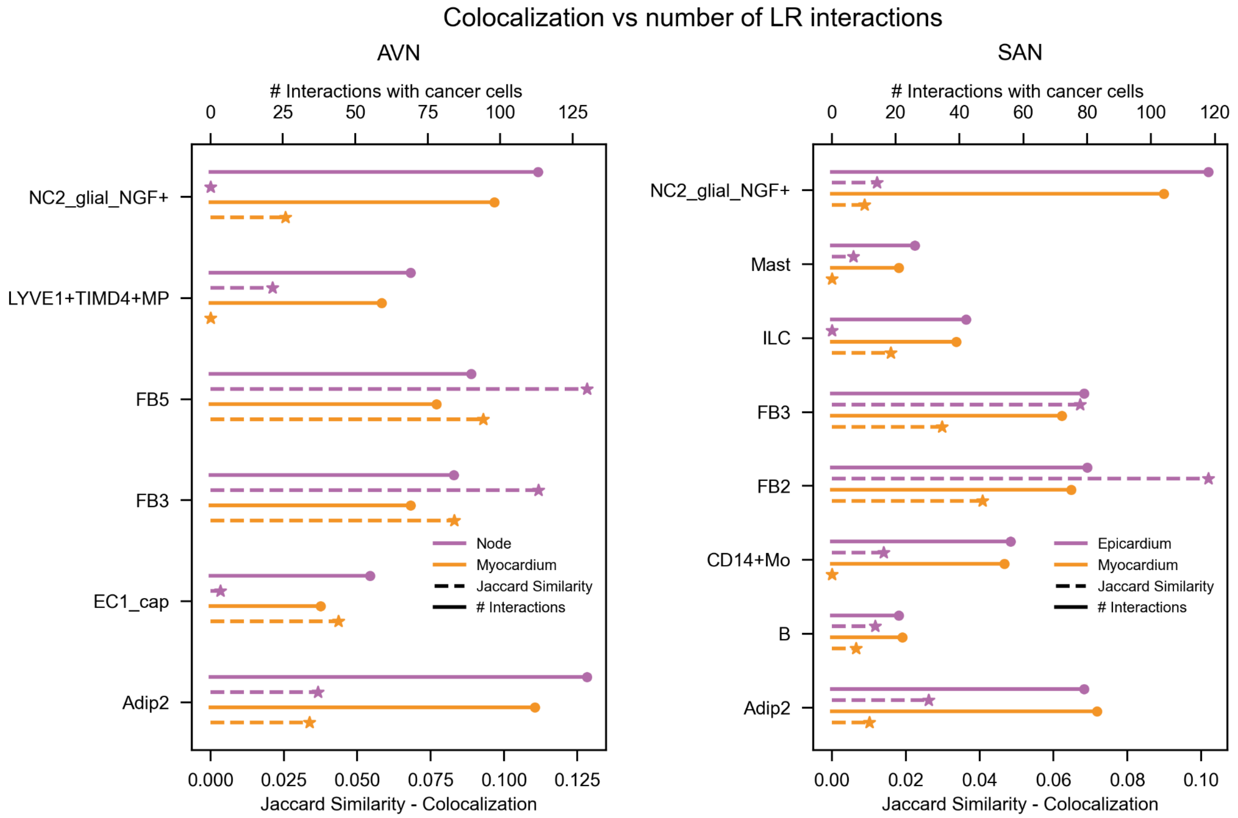

Fig S31. Comparison between colocalization and LIANA cell-cell communication results in AVN and SAN slides. This figure presents a comparative analysis of colocalization results and the number of ligand-receptor interactions predicted between histologically-defined fibroblasts and cells in the y-axis. Plain line represents the number of significant ligand-receptor interactions identified by LIANA, utilizing the consensus score. Dotted line illustrates the degree of colocalization, measured as the Jaccard similarity index between the spot profiles of fibroblasts and y-axis cells (based on the presence or absence of cells in each spot). For visualization purposes, we selected the extreme cases, those with the highest and lowest levels of colocalization and interaction frequencies.

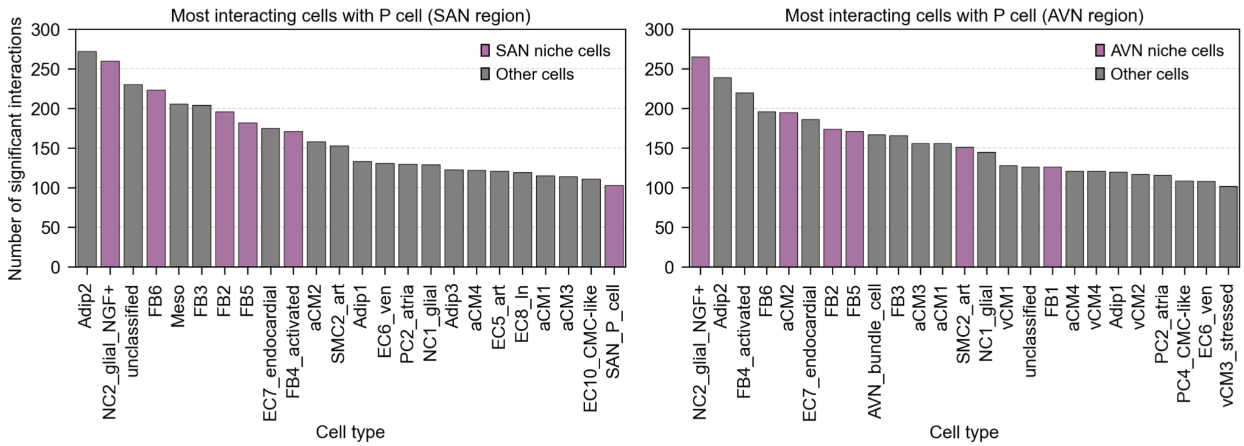

Fig S32. Most interacting cell types with P cells in the SAN / AVN region based on CellPhoneDB results without spatial information. Bar Plot showing the number of significant predicted interactions (≥100) between P cells and other cell types in the SAN / AVN region. Cell types previously identified as components of the SAN niche or AVN niche in the original publication are highlighted in purple; all others are shown in gray. SAN niche cell types: SAN_P_cell, FB2, FB4_activated, FB5, FB6, NC2_glial_NGF+ and LYVE1+IGF1+ MP. AVN niche cell types: AVN_P_cell, aCM2, FB1, FB2, FB5, SMC1_basic, SMC2_art, NC2_glial_NGF+, LYVE1+IGF1+ MP and mast.

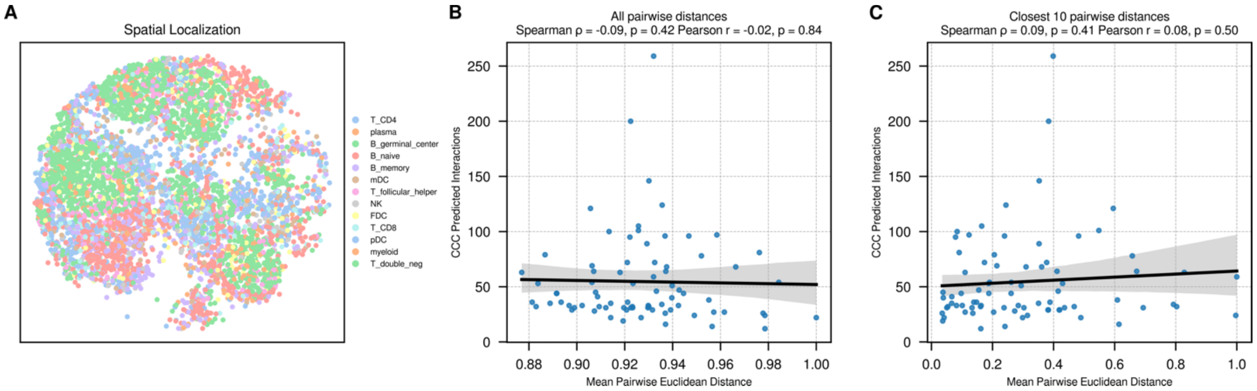

Fig S33. Analysis of the tonsil Slide-tags study. A. localization of the different cells, colored by cell type, in the tonsil. **B**. Correlation between physical proximity and predicted cell-cell communication (CCC) in the Slide-tags tonsil dataset. Each point represents a pair of cell types, plotted by their average pairwise Euclidean similarity (computed across all nuclei pairs from the two cell types and normalized over the largest distance in the tonsil slide) and the number of significant ligand-receptor interactions inferred using CellPhoneDB. Spearman and Pearson correlations were calculated between all the pairs. **C**. Same as B but using the average distance to the closest 10 cells of each cell type, to ensure that the real proximity between pairs of cells is captured.

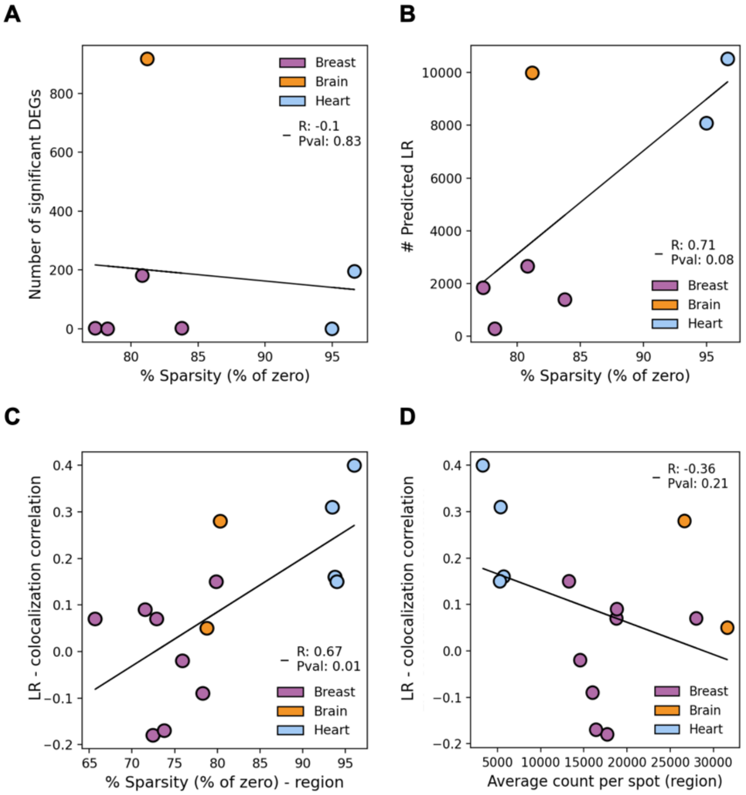

Fig S34. Correlation between spatial transcriptomics sparsity and downstream analysis metrics. **A**. Pearson correlation between slide-level sparsity (percentage of zero values) and the number of differentially expressed genes (DEGs) between defined regions. No significant correlation was found (R = -0.1, p = 0.83). **B**. Pearson correlation between slide-level sparsity and the total number of predicted ligand-receptor interactions (LR). A marginal positive correlation is observed (R = 0.71, p = 0.08), suggesting potential inflation of CCC predictions in sparse datasets. **C**. Pearson correlation between region-level sparsity and the correlation between CCC predictions and spatial colocalization. A significant positive correlation was found, suggesting that sparse slides may inflate spatial-CCC agreement (R = 0.67, p = 0.01). **D**. Pearson correlation between the average UMI count per spot and CCC-colocalization correlation. Although not statistically significant (R = -0.36, p = 0.21), the negative trend supports the idea that higher-quality data does not necessarily strengthen spatial coherence in CCC predictions (R = -0.36, p = 0.21). Color codes indicate tissue type: Breast (purple), Brain (orange), and Heart (blue).
